# Biostimulant Effects of Glutacetine® and Its Derived Formulations Mixed With N Fertilizer on Post-heading N Uptake and Remobilization, Seed Yield, and Grain Quality in Winter Wheat

**DOI:** 10.3389/fpls.2020.607615

**Published:** 2020-11-13

**Authors:** Victor Maignan, Benoit Bernay, Patrick Géliot, Jean-Christophe Avice

**Affiliations:** ^1^Normandie Univ, UNICAEN, INRAE, UMR EVA, SFR Normandie Végétal FED4277, Esplanade de la Paix, Caen, France; ^2^Via Végétale, Le Loroux-Bottereau, France; ^3^Plateforme Proteogen, SFR ICORE 4206, Université de Caen Normandie, Esplanade de la Paix, Caen, France

**Keywords:** plant biostimulants, nitrogen fertilizer, *Triticum aestivum*, nitrogen remobilization, senescence, phytate, iron bioavailability, grain quality

## Abstract

Biostimulants could play an important role in agriculture particularly for increasing N fertilizer use efficiency that is essential for maintaining both yield and grain quality in bread wheat, which is a major global crop. In the present study, we examined the effects of mixing urea–ammonium–nitrate fertilizer (UAN) or urea with five new biostimulants containing Glutacetine® or its derivative formulations (VNT1, 2, 3, and 4) on the physiological responses, agronomic traits, and grain quality of winter wheat. A first experiment under greenhouse conditions showed that VNT1, VNT3, and VNT4 significantly increased the seed yield and grain numbers per ear. VNT4 also enhanced total plant nitrogen (N) and total grain N, which induced a higher N Harvest Index (NHI). The higher post-heading N uptake (for VNT1 and VNT4) and the acceleration of senescence speed with all formulations enabled better nutrient remobilization efficiency, especially in terms of N mobilization from roots and straw toward the grain with VNT4. The grain ionome was changed by the formulations with the bioavailability of iron improved with the addition of VNT4, and the phytate concentrations in flour were reduced by VNT1 and VNT4. A second experiment in three contrasting field trials confirmed that VNT4 increased seed yield and N use efficiency. Our investigation reveals the important role of these new formulations in achieving significant increases in seed yield and grain quality.

## Introduction

Bread wheat (*Triticum aestivum* L.) is the largest primary commodity and a major global cereal crop, with production of approximately 700 Mt annually that provides 19% of the world’s total available calories (FAOSTAT, 2020). China is the primary producer (112 Mt) before India (78 Mt), the United States (58 Mt), Russia (49 Mt), and France (36 Mt). Farm-gate yields worldwide have increased over the past half century to the current 3 t ha^–1^ (Hawkesford, 2017). In highly productive temperate areas, for example, in France, farm-gate yields are 9 t ha^–1^, but with an apparent plateauing in recent years (Brisson et al., 2010). Due to economic and ecological factors, there is a trend in Europe to limit chemical inputs for wheat, especially N (Boisson et al., 2005), despite it being essential for both maximal yield and optimal quality in terms of protein content. Seed yield and grain protein concentration are two major challenges in winter wheat, as these traits are dominant determinants of the economic value of the harvested product (Pan et al., 2020). Protein content influences price, especially due to its impact on the rheological qualities of flour (Nadaud et al., 2015). However, there is a strong negative relationship between seed yield and grain N concentration (Bogard et al., 2010; Brisson et al., 2010; de Oliveira Silva et al., 2020). In wheat, 60–90% of the grain N derives from remobilization of stores accumulated in vegetative organs prior to flowering. In contrast, the N taken up during the post-flowering period represents between 5 and 40% of the total grain N under field conditions (Oscarson, 2000). The relative contribution of this later N source to grain N is strongly influenced by the environment and especially by the availabilities of N and water in the soil (Miller et al., 2007). Under controlled conditions where environmental constraints are minimized, wheat has the ability to take up N until close to grain maturity (Glass, 2009; Masclaux-Daubresse et al., 2010). It is also well documented that post-flowering N uptake has a strong impact on the grain N concentration in wheat (Oscarson, 2000; Taulemesse et al., 2015). Interestingly, focusing on the post-flowering period, many studies have been undertaken on senescence processes in wheat and their importance in N use efficiency (NUE) (Gregersen et al., 2008; Distelfeld et al., 2014; Gaju et al., 2014; Hebbar et al., 2014). Indeed, nutrient allocation is a dynamic phenomenon, achieved by nutrient recycling and linked to processes of development including senescence and remobilization (Hawkesford et al., 2018). During senescence, proteins are degraded, and nutrients are remobilized from senescing organs toward developing grain (Gregersen et al., 2008). A balance between senescence timing and speed, grain nutrient content, nutrient use efficiency, and seed yield needs to be considered to improve wheat production and its quality components. In this regard, the effectiveness of remobilization strongly influences performance and quality, particularly grain protein and mineral nutrient contents, which are important health and quality attributes of the seeds. The nutrient remobilization efficiency is an essential component of nutrient use efficiency, an important sustainability trait (Avice and Etienne, 2014; de Oliveira Silva et al., 2020).

N is essential to synthesize seed storage proteins in wheat, such as glutenin or gliadins (Dupont et al., 2006). Because they contribute to baking quality, a balanced mixture of these components is crucial (Barak et al., 2013). However, these proteins are also related to celiac disease (Gell et al., 2017; Perrin et al., 2019) and especially the α-gliadins (Sollid et al., 2012; Zörb et al., 2013). A downregulation of these proteins, while still maintaining dough proprieties, might be a solution for celiac disease patients (Altenbach et al., 2020). Beyond N, mineral nutrients in grain act as a source of micronutrients in the human diet, among which deficiencies in key minerals including calcium (Ca), magnesium (Mg), copper (Cu), iron (Fe), and zinc (Zn) have prompted efforts to increase their concentrations in seeds (Kutman et al., 2010; Hussain et al., 2012; Ramzani et al., 2016; Rehman et al., 2018; Wang et al., 2020). In the last few decades, it has been well established that wheat is a crucial source of Fe and Zn for humans (Uauy et al., 2006; Ciccolini et al., 2017; Singh et al., 2018), but the concentrations of these minerals in flour are generally low (Sobolewska et al., 2020), and their bioavailability is limited by high phytate content (Ficco et al., 2009; Xue et al., 2015). Phytic acid is linked to mineral deficiency because of its high affinity with minerals, mainly Fe, Zn, Ca, and Mg (Ficco et al., 2009). Therefore, fertilization strategies improving the nutritional value of wheat grain might allow production of mature seeds with high concentrations of beneficial micronutrients and low contents of phytate (Safar-Noori et al., 2018) or toxic micronutrients, such as cadmium (Cd). Indeed, Cd is toxic for humans and notably damages kidney function, even at very low concentrations (Järup et al., 1998; Huang et al., 2020). Many areas of arable soil in the world are contaminated by Cd through the use of sludges, phosphate fertilizers, or irrigation water containing Cd (McGrath et al., 2001). In this regard, it is very important to reduce the accumulation of Cd in wheat grain to below the maximum concentration of 0.2 μg Cd g^–1^ allowed in Europe (Commission Regulation, 2006). One way to minimize the Cd loaded into wheat grain is to adapt mineral nutrition (Sarwar et al., 2010; Yan et al., 2018), such as N (Gray et al., 2002), phosphorus (Gao and Grant, 2012), silicon (Rizwan et al., 2012), or micronutrients (Huang et al., 2020). Thus, management of nutrient inputs that impact senescence processes could affect the loading of Cd into seeds.

One way to reduce N fertilizers without dramatically affecting grain yields is to improve the N recycling and remobilization performance of plants. Enhanced-efficiency fertilizers, such as those containing nitrification inhibitors and urease inhibitors, have been developed to increase NUE and reduce N losses by increasing the congruence between N supply and crop N demand (Abalos et al., 2014). However, these inhibitors could also have a negative impact on plant metabolism (Artola et al., 2011; Zanin et al., 2016) and affect the activity of soil microorganisms (Guardia et al., 2018; Castellano-Hinojosa et al., 2020). More recently, solutions classified as plant growth stimulants have been studied (Calvo et al., 2014; Colla et al., 2014; Chiaiese et al., 2018; Popko et al., 2018; Paul et al., 2019). A plant biostimulant is any substance or microorganism applied to plants with the aim to enhance nutrition efficiency, abiotic stress tolerance, or crop quality traits, regardless of its nutrient content (du Jardin, 2015). The role of biostimulants is to control and accelerate the life processes of plants, increase the resistance to stress, and stimulate their development (Calvo et al., 2014). These products are also safe for the environment and contribute to sustainable, high-output low-input crop production (du Jardin, 2015). Their application helps to reduce the amount of chemicals used in agriculture. Among biostimulants, plant extracts, amino acids, and beneficial elements have been widely studied (Rouphael and Colla, 2020). Biostimulants based on proteins or amino acids have enhanced N metabolism (Di Mola et al., 2020; Hassan et al., 2020), crop yield, grain characteristics, and the content of macro- and micronutrients in winter wheat (Popko et al., 2018). Regarding quality traits, plant biostimulants could affect durum wheat grain properties, changing the grain proteome (Pichereaux et al., 2019). Mineral interactions are also known to be essential for improving mineral use efficiency. Moreover, it is well documented that the actions of N and S fertilizers are strongly linked and impact seed yield components and quality (Poisson et al., 2019; Guerrini et al., 2020). Other elements, such as Ca (Liu et al., 2020), chloride (Cl), or boron (B), facilitate N assimilation (López-Lefebre et al., 2000; Bielski et al., 2020) and stimulate N metabolism (Wang et al., 2019a) even under abiotic stresses (Arshi et al., 2006; Das et al., 2016; Kaczmarek et al., 2016). Designing and developing new biostimulants or enhanced-efficiency fertilizers is a crucial process that requires an accurate testing of the product’s effects on the morpho-physiological traits of plants and a deep understanding of their mechanism of action.

The aim of the present study was to determine the effect of new additives to N fertilizers on NUE, yield, and grain quality in winter wheat. To address this goal, two experiments were carried out for determination of relevant impacts of news additives on physiological, agronomic, and grain quality parameters. Firstly, an experiment under semi-hydroponic conditions was performed in a greenhouse, using local fertilization practices with the five organo-mineral additives (Table 1 for more details about the composition of formulations) mixed with N fertilizers (to form an enhanced-efficiency fertilizer) and applied at three different growth stages (GS) to achieve the equivalent of 150 kg N ha^–1^. A second experiment was carried out under three contrasting field conditions in order to test the impact of the more promising formulation identified in experiment 1 (i.e., the formulation VNT4) mixed with urea or UAN solution (50% urea, 25% NO_3_^–^, 25% NH_4_^+^).

**TABLE 1:** The chemical composition of the tested formulations.

Component	Glutacetine®	VNT1	VNT2	VNT3	VNT4
Type of application	Foliar	Soil	Soil	Soil	Soil
Glutamic acid (%)	3.6	19.6	10	3.7	3.6
Organic acids (%)	7.4	0	7	5.5	7.4
Total soluble sugars (g L^–1^)	22.2	26.5	31.0	25.4	34.4
**Elements (%)**					
Cl	19.8	12.5	17.4	12.0	18.2
Ca	15.6	11.5	10.1	14.5	13.4
C	6.26	9.43	14.51	15.53	11.49
N	0.76	0.72	1.36	1.18	0.88
K	0.31	0.26	0.23	0.33	0.29
Na	0.31	0.16	0.14	0.19	0.18
Mo	0.15	0.19	0.18	0.19	0.18
**Elements (ppm)**					
S	300	130	120	150	140
B	30	20	20	20	20
Mg	19	8	7	13	11
Si	14	11	9	15	11
P	9.5	9	9.9	10.3	10.2
Cu	2.6	1	0.8	1.1	1.1
Ni	1.8	0.6	0.5	0.6	0.6
Co	0.6	0.5	0.1	0.1	0.4
Zn	0.3	0.5	1.1	1.8	3.5
Se	0.08	0.07	0.06	0.04	0.06

## Materials and Methods

### Experiment Under Greenhouse Conditions (Experiment 1)

#### Experimental Design and Tissue Sampling

Winter wheat (*T. aestivum* L., cv. Récital) was sown in germination trays filled with a sand and vermiculite mixture (2v:1v) supplied with 25% Hoagland nutrient solution (1.25 mM Ca(NO_3_)_2_, 4H_2_O; 1.25 mM KNO_3_; 0.5 mM MgSO_4_; 0.25 mM KH_2_PO_4_; 0.2 mM EDTA, 2NaFe, 3H_2_O; 14 μM H_3_BO_3_; 5 μM MnSO_4_; 3 μM ZnSO_4_; 0.7 μM (NH_4_)_6_Mo_7_O_24_; 0.7 μM CuSO_4_; 0.1 μM CoCl_2_) and placed for 2 weeks under greenhouse conditions (16 h light at 20°C, 8 h dark at 16°C). Natural light was supplied by high-pressure sodium lamps (Philips, MASTER GreenPower T400W). After emergence, plants were vernalized in a growth chamber (6°C, photoperiod 8 h) for 6 weeks. Following vernalization, plants were transplanted into PVC tubes (8 cm diameter × 33 cm high) filled with a sand:perlite mixture (1v:1v) for semi-hydroponic culture. The bottom of each pot was perforated to allow excess nutrient solution to drain. Two plants were transplanted into each tube, and tubes were placed vertically in containers (five tubes per container) in order to obtain a plant density of 250 plants m^–2^, which is comparable to the density usually observed in the field under local agronomic practices.

Containers were placed in a heated greenhouse (16 h light at 20°C, 8 h dark at 16°C) and completely randomized. Natural light was supplied by high-pressure sodium lamps (Philips, MASTER GreenPower T400W) with a PAR of 350 μmol m^–2^ s^–1^ at canopy height. During the first 15 days, each tube received 30 ml of a 25% Hoagland nutrient solution three times a day *via* an automated micro-irrigation system coupled with a percolate recycling system. After tillage, each tube received nutrient solution without NO_3_^–^ (1.25 mM CaCl_2_, 2H_2_O; 1.25 mM KCl; 0.5 mM MgSO_4_; 0.25 mM KH_2_PO_4_; 0.2 mM EDTA, 2NaFe, 3H_2_O; 14 μM H_3_BO_3_; 5 μM MnSO_4_; 3 μM ZnSO_4_; 0.7 μM (NH_4_)_6_Mo_7_O_24_; 0.7 μM CuSO_4_; 0.1 μM CoCl_2_). UAN solution (50% urea, 25% NO_3_^–^, 25% NH_4_^+^) was split into three inputs: 50 units (UN: kg N ha^–1^) at the tillering stage (GS29), 80 units at the 2-node stage (GS32), and 20 units at the heading stage (GS59). Five additives to fertilizers (Glutacetine® and VNT #1 to #4 provided by Via Végétale, Le Loroux-Bottereau, France) were tested in the present study and then compared with a control (N fertilizer alone). These formulations contain organo-mineral complexes with different compositions (Table 1). Glutacetine® was provided as a powder, and 3.5 kg was dissolved in 5 L (mother solution) to be sprayed on 1 ha as recommended by Via Végétale. After dilution of 200 μl of mother solution in 25 ml ultrapure water, this formulation was applied using a sprayer on 5 tubes (10 plants). Finally, each plant received 2.5 ml of Glutacetine^®^ formulation only during the last N fertilizer application (the first two applications were added to the substrate without Glutacetine®). There were three doses of the formulations applied: the first dose was 83 ml UN^–1^, the second was 166 ml UN^–1^, and the third dose was the equivalent of 3 × 5 L ha^–1^ (dose 3 was tested only with VNT4). The chlorophyll levels in vegetative parts were measured each week from the tillering (GS29) to maturity (GS89) stages with an optical sensor system (Multiplex® fluorometer, Force A, Orsay, France).

Three sampling dates were chosen during the growth cycle. The first destructive sampling took place at emergence of the last visible leaf (GS39), the second at heading (GS59), and the last at maturity (GS89). Each sampling was made at the same time of the day (1–2 h after the start of the light period). On each sampling date, three tubes (six plants) of each treatment were collected for physiological measurements and elemental analyses. Each tube (two plants) was considered as a biological replicate. At the intermediate harvest, the number of spikes was measured. The plants were then separated into three fractions: roots, vegetative aerial parts, and reproductive organs. All samples were dried in an oven (60°C) for 48 h before dry weight (DW) measurement. Dry samples were ground to a fine powder using the Retsch MM200 mixer mill (Eragny sur Oise, France) for elemental, phytate, and proteomic analyses.

#### Determination of Leaf Senescence Speed and Components of N Use Efficiency

Senescence speed was expressed as the slope of decrease in chlorophyll index between the end of flowering (GS69) and maturity (GS89) stages.

The relative concentrations of total N in the different tissues were determined using a C/N/S analyzer (EA3000; EuroVector, Milan, Italy) connected to a continuous flow isotope mass spectrometer (IRMS, Isoprime; GV Instruments, Manchester, United Kingdom). Based on the N contents, the N Harvest Index (NHI) and N use efficiency (NUE) were calculated. The NHI corresponds to the ratio between the N amounts in seeds and the total N amount in the plant at the final harvest. NUE is expressed as the grain DW produced per gram of N provided by fertilizers. Post-heading N uptake was calculated by the difference between total N at the maturity stage and total N at the heading stage. The level of N remobilization in the grain was estimated as the percentage of N remobilized in roots and vegetative parts between the heading stage and the maturity stage: %N remobilized = [(Total organ N at heading/Total organ N at maturity)/(Total organ N at heading) × 100]. When calculation of the percentage of N remobilized produced a negative value, N remobilization was considered as 0%.

#### Seed Yield Components

After the last harvest of winter wheat, the number of grains per spike, thousand grain weight (TGW in g), the harvest index (grain DW divided by total DW), specific weight (grain density, kg hl^–1^), and protein content (*N* × 5.7%) was determined. Fruiting efficiency was also calculated as the number of grains per unit of spike DW at anthesis (Slafer et al., 2015).

#### Components of Grain Quality

##### Ionomic analysis and phytate contents in seeds

K, P, S, Mg, Ca, Na, Fe, Zn, Mn, Cu, Mo, B, Cd, and Se in the wheat grain were quantified by inductively high-resolution coupled plasma mass spectrometry (HR ICP-MS; Thermo Scientific, Element 2^TM^) with prior microwave acid sample digestion (Multiwave ECO; Anton Paar, Les Ulis, France) (800 μl of concentrated HNO_3_, 200 μl of H_2_O_2_, and 1 ml of Milli-Q water for 40 mg DW). For the determination by HR ICP-MS, all the samples were spiked with two internal-standard solutions of gallium and rhodium for final concentrations of 10 and 2 μg L^–1^, respectively, diluted to 50 ml with Milli-Q water to obtain solutions containing 2.0% (v/v) nitric acid, and then filtered at 0.45 μm using a Teflon filtration system (Filtermate; Courtage Analyses Services, Mont-Saint-Aignan, France). Quantification of each element was performed using external standard calibration curves. The amount of a given element, *E*, was calculated from DW and element concentration (%*E* as % of DW) as: *E* = (%*E* × DW)/100.

Because the level of phytate is considered as a quality trait of wheat grain (the lower the better), phytate content was performed following the methodology proposed by Haug and Lantzsch (1983). The phytic acid assay was based on precipitation of ferric phytate and measurement of iron (Fe) remaining in the supernatant. This method was slightly modified in the present study. About 40 mg of ground wheat grain was used for extraction of phytic acid in 2 ml of 0.2 N HCl for 2 h and was then centrifuged at 4,600 × *g* for 20 min. The supernatant (100 μl) was treated with 900 μl of 0.2 N HCl and 2 ml of ferric solution (NH_4_Fe(SO_4_)_2_, 12 H_2_O) in a boiling water bath for 30 min. After cooling, 1 ml of supernatant was treated with 1.5 ml 2,2′-bipyridine solution to measure the Fe remaining in the supernatant. The resulting pink color was read at 524 nm from a standard curve (0, 25, 50, 100, and 200 mg L^–1^).

##### Seed proteomic analysis

Proteins were extracted from 25 mg of wholemeal flour milled for 5 min at 4°C in cold KCl (50 mM Tris–HCl, 100 mM KCl, 5 mM EDTA, pH 7.8). The sample was then centrifuged for 15 min (12,000 rpm, 4°C), and supernatant 1 was collected. Then, 400 μl of SDS sample buffer (2% SDS, 10% glycerol, 50 mM DTT, 40 mM Tris–HCl, pH 6.8) was added to the pellet for 1 h of incubation at ambient temperature. After centrifugation, the supernatant was collected, and 40 μl (10% v/v) of cold TCA 72% was added for 2 h of incubation at 4°C. After centrifugation, the resulting pellet was washed three times in ice-cold acetone, then dried at room temperature, and finally added to supernatant 1 for Bradford analysis and SDS-PAGE electrophoresis (20 μg protein). For nano-LC fragmentation, protein or peptide samples were first desalted and concentrated onto μC18 Omix pipette tips (Agilent) before analysis. The chromatography step was performed on a NanoElute (Bruker Daltonics) ultra-high pressure nanoflow chromatography system. Peptides were concentrated onto a C18 pepmap 100 (5 mm × 300 μm i.d.) precolumn (Thermo Scientific) and separated at 50°C onto a reversed phase Reprosil column (25 cm × 75 μm i.d.) packed with 1.6 μm C18 coated porous silica beads (IonOpticks). Mobile phases consisted of 0.1% formic acid, 99.9% water (v/v) (A), and 0.1% formic acid in 99.9% ACN (v/v) (B). The nanoflow rate was set at 400 nl/min, and the gradient profile was as follows: from 2 to 15% B within 60 min, followed by an increase to 25% B within 30 min and further to 37% within 10 min, and followed by a washing step at 95% B and re-equilibration.

Mass spectrometry (MS) experiments were carried out on a TIMS-TOF pro mass spectrometer (Bruker Daltonics) with a modified nano electrospray ion source (CaptiveSpray; Bruker Daltonics). The system was calibrated each week, and mass precision was better than 1 ppm. A 1,600 spray voltage with a capillary temperature of 180°C was typically employed for ionizing. MS spectra were acquired in the positive mode in the mass range from 100 to 1,700 m/z. In the experiments described here, the mass spectrometer was operated in PASEF mode with exclusion of single charged peptides. A number of 10 PASEF MS/MS scans were performed for 1.16 s from charge range 2–5. The fragmentation pattern was used to determine the sequence of the peptide. Database searching and label-free quantification (LFQ) were performed using Peaks X software. A Uniprot *T. aestivum* (including 105,061 entries) database was used. The variable modifications allowed were as follows: C-carbamidomethyl, C-propionamide, K-acetylation, methionine oxidation, and deamidation (NQ). “Trypsin” was selected as semispecific. Mass accuracy was set to 20 ppm and 0.05 Da for MS and MS/MS mode, respectively. Data were filtered according to a peptide false discovery rate (FDR) of 1%, ≥ 2 unique peptides per protein, and the elimination of protein redundancy on the basis of proteins being evidenced by the same set or a subset of peptides. For LFQ, the Peaks Q method was used with TIC as the normalization factor and quality > 5. Peptide detection was required in at least two samples per group, and modified forms were excluded. A 1.5-fold increase in relative abundance and a significance of ≥ 20 were used to determine enriched proteins. The mass spectrometry proteomics data have been deposited to the ProteomeXchange Consortium *via* the PRIDE (Perez-Riverol et al., 2019) partner repository with the dataset identifier PXD021512.

### Experiment Under Field Conditions (Experiment 2)

During autumn 2018, three different cultivars of winter wheat (*T. aestivum* L., cv. Sacramento, Libravo, and Chevignon) were sown in three different sites in Normandy, France (see Supplementary Table 1 for more details), which belong to the oceanic temperate region. Plot size was 24 m^2^ (12 m × 2 m), and three replicates (plots) were used per treatment on each site. The plots were randomized in three main blocks. During April 2019–June 2019, systematic applications of plant protection treatments were carried out. Weather conditions (average temperature and total rainfall) were from 11.9 to 12.4°C and from 588 to 611 mm between September 2019 and August 2020 (Supplementary Figure 1). Previous crops and soil properties were very different in each site (Supplementary Table 1).

On each site, all plots were fertilized with 30 kg S ha^–1^ at the tillering stage (BBCH 29, Lancashire et al., 1991). N fertilization was intended to replicate farmer practice and was calculated using the method (so called “Méthode du Bilan”) described by the French committee COMIFER (Comité français d’étude et de développement de la fertilisation raisonnée, 2000). This method measures the amount of mineral N in soil in February in order to estimate total N soil supply. With the estimation of total plant needs, the method allows to apply N dose close to the crop demand (Supplementary Table 2). Two different N fertilizers were used: urea 46% and ammonium nitrate urea solution 39% (UAN). N fertilization dose was applied at 152, 210, and 152 kg N ha^–1^ for sites 1, 2, and 3, respectively (Supplementary Table 2). The first fertilization was applied at the tillering stage (BBCH 21), the second at the stem elongation stage (BBCH 31), and the rest at the last leaf stage (BBCH 39, Supplementary Table 2). For the last N application, we followed the new N requirement indicators (coefficient “bq”) to grow each bread wheat cultivar with a dual objective of optimum yield and grain protein contents in line with the market requirements (Cohan et al., 2019). For the biostimulant treatment, 5 L ha^–1^ of VNT4 was mixed with each N application and applied to the soil to achieve 15 L ha^–1^ in each site. Ear number was counted on each plot of the three sites, and grains and straws were collected using a combined harvester. All samples were dried (60°C) for 48 h and then ground to a fine powder for agronomic, physicochemical, and N analyses.

After harvesting of winter wheat, 1,000 grain weight (TGW, g), grain number per spike, and moisture (%) were determined. Yield was then calculated with a standard moisture (15%) and NUE according to Moll et al. (1982) using the following equations: NUE (kg kg^–1^) = Grain yield (kg ha^–1^)/N supply (kg ha^–1^). The relative concentrations of total N in the different tissues were determined, and N exported by grains was then calculated.

### Statistical Analysis

For individual treatments, the greenhouse experiment (Exp. 1) was performed with three independent biological replicates consisting of one tube (1 replicate = 2 plants per tube). Data are represented as mean ± standard error (SE) for *n* = 3. After verifying compliance of normality using Shapiro–Wilk test, analysis of variance (ANOVA) and Fisher’s test (R software) were employed to analyze all the data and marked by different letters when significantly different (*p* < 0.05). For the field experiment (Exp. 2), all statistical analyses were performed in R Statistical Software version 3.3. An ANOVA analysis was used to evaluate the effect of site, fertilizer, and VNT4 (*n* = 3). Then, Fisher’s test was employed to analyze all the dates and marked by different letters when significantly different (*p* < 0.05).

## Results

### Effect of the Formulations Under Greenhouse Conditions (Exp. 1)

In the present study, we tested the effect of N fertilizers containing different formulations of Glutacetine®-based biostimulants on growth, yield, N partitioning, and grain quality in winter wheat. The analyses of variance showed strong effects of the formulations, the dose, and an interaction between the treatment and the dose depending on the parameters (Supplementary Table 3). That is why, we performed a Fisher’s test to analyze all the data and marked by different letters when significantly different at 5%.

#### Effect of the Formulations on Biomass, Seed Yield, and Grain Parameters

**FIGURE 1:**
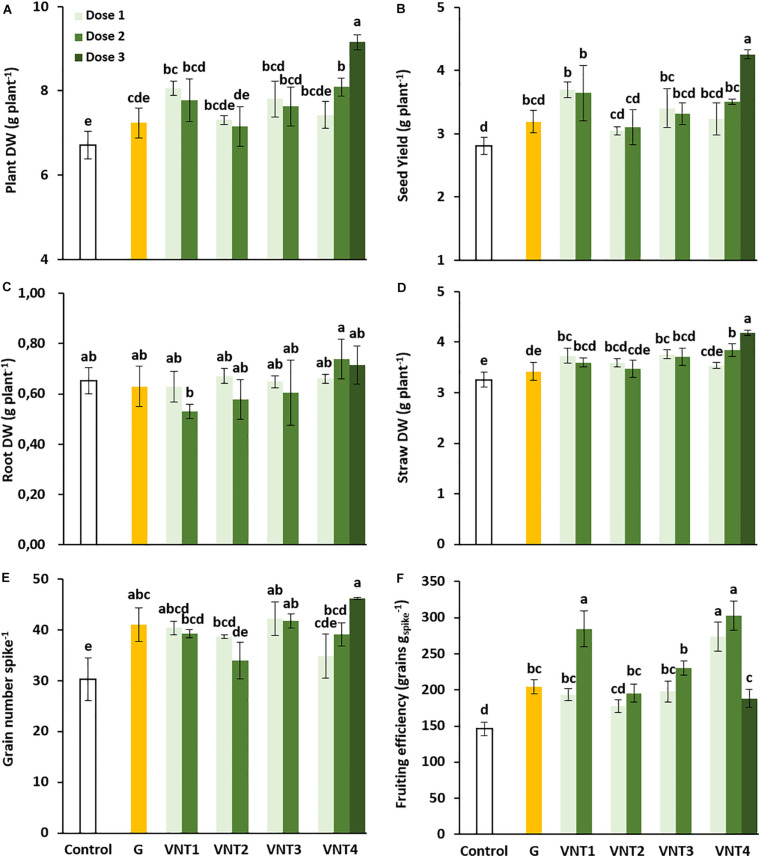
Effect of biostimulant formulations mixed with N fertilizer on plant dry weight (DW), seed yield, root DW, straw DW, grain number per spike, and fruiting efficiency in wheat (*Triticum aestivum* L.). **(A)** Plant dry weight, **(B)** seed yield, **(C)** root dry weight, **(D)** straw dry weight, **(E)** grain number per spike, and **(F)** fruiting efficiency. Plant culture was carried out under semi-hydroponic conditions on a sand/perlite (1/1) substrate (see the Materials and Methods section for details). N was provided at the tillering (eq. 50 kg N ha^–1^), the 2 nodes (eq. 80 kg N ha^–1^), and the heading stages (eq. 20 kg N ha^–1^). Glutacetine® (G) was mixed with N fertilizer at the heading stage at a dose of 5 L ha^–1^ and applied as a foliar treatment. Four other formulations (VNT1, VNT2, VNT3, and VNT4, see Table 1 for composition of each) were mixed with N fertilizer at different doses: dose 1 was 83 ml kg N^–1^, dose 2 was 166 ml kg N^–1^, and dose 3 was the equivalent of 3 × 5 L ha^–1^. Dose 3 was tested only with VNT4. Plants were harvested at maturity (GS89). Bars indicate mean ± SE. Different letters denote significant differences according to Fisher’s test (*p* < 0.05; *n* = 3).

To determine the physiological response of plants to the formulations tested (Table 1), the growth response was evaluated at GS39, GS59, and GS89. There was no consistent change at GS39 and GS59 (Supplementary Figure 2), but at GS89, VNT1, VNT3, and VNT4 (doses 2 and 3) improved plant DW (6.72 g DW plant^–1^ for control versus 9.16 g DW plant^–1^ for VNT4-D3, Figure 1A) which is due to a better seed yield for all formulations compared with the control plants (Figure 1B). Interestingly, seed yield increased significantly with VNT1, VNT3-D1, and VNT4-D2 and especially with VNT4-D3 (+ 51.7%, Figure 1B). Grain yields were high (equivalent to 75 and 114 q ha^–1^ for the two extreme treatments), which was probably due to the semi-hydroponic growth conditions. However, root DW was identical irrespective of the formulation (Figure 1C). In contrast, straw biomass, which correlated with grain yield (*r*^2^ = 0.65), ranged from 3.26 g DW plant^–1^ for the control to 4.19 g DW plant^–1^ for VNT4 at dose 3. Indeed, VNT1, VNT2-D1, VNT3, and VNT4 (doses 2 and 3) significantly increased straw DW (Figure 1D), which contributed to improve plant DW (Figure 1A).

The improvement in seed yield was related to the increase in grain number per spike. Indeed, except for VNT2-D2 and VNT4-D1, the number of grains per spike was significantly higher for all formulations with a maximum observed for VNT4-D3 plants (46 vs 30.2 grains per spike in the control group) (Figure 1E). Very interestingly, fruiting efficiency was also improved in response to all formulations, excluding VNT2 at the first dose (Figure 1F), suggesting that these formulations could increase wheat fertility during the flowering period. In the present study, the spike number per plant did not show consistent change (Table 2). Nevertheless, due to the better grain number per spike, the grain number per plant was increased by all formulations (except VNT2-D2, Table 2). Compared with the control, Glutacetine^®^, VNT2-D1, and VNT3 reduced the TGW, whereas VNT1-D2, VNT2-D2, and VNT4 (doses 1 and 2) improved the specific weight (Table 2). The harvest index lies between 0.42 and 0.47, which is close to values observed under field conditions (Barraclough et al., 2010). A significant increase in the harvest index was also observed in response to VNT1 (doses 1 and 2) and VNT4-D3 (+ 9.8, + 11.7, and + 11.2%, respectively, Table 2).

**TABLE 2:** Influence of formulations on harvest index, spike number per plant, grain number per plant, 1,000 grain weight (TGW), specific weight, protein content, and N use efficiency in wheat (*Triticum aestivum* L.) (Experiment 1).

	Harvest index	Spike number/plant	Grain number/plant	1,000 grain weight (g)	Specific weight (kg hl^–1^)	Protein content (%)	N use efficiency
**Control**	0.42 ± 0.003b	3.00 ± 0.35	88.67 ± 3.05e	33.02 ± 1.77a	70.75 ± 1.62b	14.17 ± 0.23a	6.97 ± 0.24bc
**Glutacetine®**	0.44 ± 0.018ab	2.83 ± 0.20	115.83 ± 9.04bcd	28.97 ± 2.46bc	71.58 ± 0.95ab	13.22 ± 0.49ab	7.40 ± 0.38bc
**VNT1**							
Dose 1	0.46 ± 0.015a	3.17 ± 0.20	127.50 ± 4.78ab	30.37 ± 1.51abc	72.46 ± 0.90ab	12.54 ± 0.32b	8.13 ± 0.22b
Dose 2	0.47 ± 0.028a	3.00 ± 0.00	117.50 ± 13.31bcd	32.46 ± 1.38a	73.29 ± 0.72a	12.48 ± 0.37b	7.99 ± 1.02bc
**VNT2**							
Dose 1	0.42 ± 0.004b	3.00 ± 0.00	116.00 ± 0.94bcd	27.34 ± 0.45c	72.75 ± 0.72ab	13.28 ± 0.22ab	7.09 ± 0.15bc
Dose 2	0.43 ± 0.014ab	3.00 ± 0.00	102.00 ± 10.73de	32.04 ± 1.50ab	73.83 ± 1.20a	12.62 ± 0.77b	6.85 ± 0.60c
**VNT3**							
Dose 1	0.43 ± 0.017ab	3.00 ± 0.00	126.50 ± 9.82ab	28.17 ± 0.65c	72.96 ± 0.44ab	12.67 ± 0.34b	7.55 ± 0.57bc
Dose 2	0.44 ± 0.006ab	3.00 ± 0.00	125.17 ± 4.28ab	27.81 ± 1.22c	72.54 ± 1.35ab	13.03 ± 0.92ab	7.55 ± 0.19bc
**VNT4**							
Dose 1	0.43 ± 0.017ab	3.00 ± 0.00	104.50 ± 13.08ab	32.54 ± 1.93a	73.46 ± 0.54a	12.86 ± 0.64b	7.32 ± 0.80bc
Dose 2	0.43 ± 0.011ab	3.17 ± 0.20	123.17 ± 0.54abc	29.84 ± 0.39abc	73.21 ± 1.18a	12.54 ± 0.25b	7.71 ± 0.08bc
Dose 3	0.46 ± 0.002a	3.00 ± 0.00	138.50 ± 0.71a	32.10 ± 0.43ab	72.96 ± 0.57ab	12.62 ± 0.19b	9.42 ± 0.24a

*Values indicate mean ± SE. Different letters denote significant differences according to Fisher’s test (*p* < 0.05; *n* = 3).*

#### Effect of the Formulations on N Status and N Partitioning at the Whole Plant Level

**FIGURE 2:**
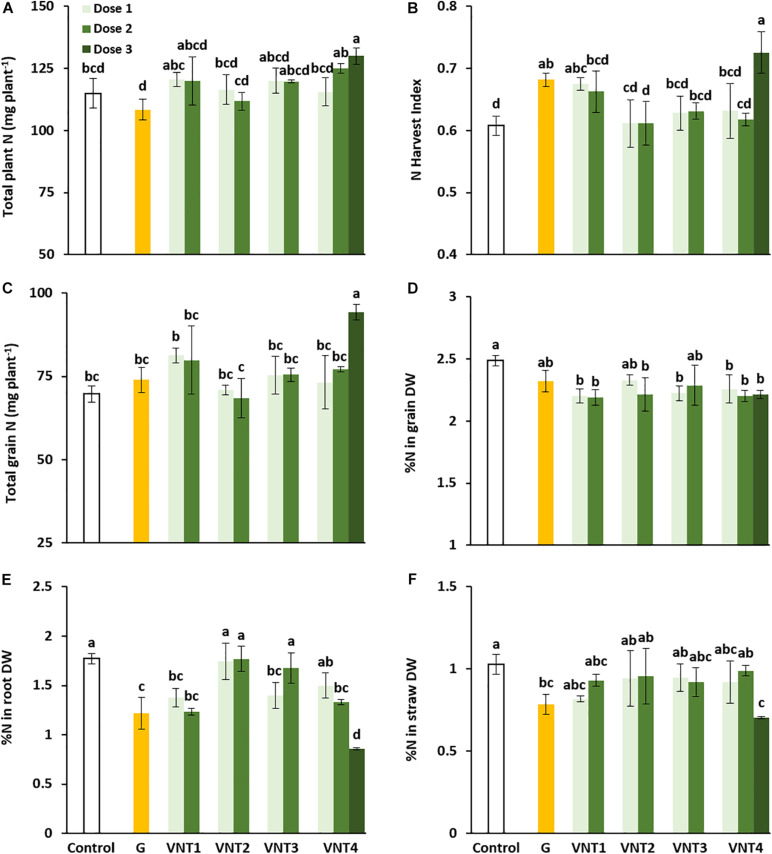
Effect of biostimulant formulations mixed with N fertilizer on N uptake and repartition in wheat (*Triticum aestivum* L.). **(A)** Total plant N, **(B)** N Harvest Index (NHI), **(C)** total grain N, **(D)** and relative N content in the grain, **(E)** roots, and **(F)** straw. Plant culture was carried out under semi-hydroponic conditions on a sand/perlite (1/1) substrate (see the Materials and Methods section for details). N was provided at the tillering (eq. 50 kg N ha^–1^), the 2 nodes (eq. 80 kg N ha^–1^), and the heading stages (eq. 20 kg N ha^–1^). Glutacetine® (G) was mixed with N fertilizer at the heading stage at a dose of 5 L ha^–1^ and applied as a foliar treatment. Four other formulations (VNT1, VNT2, VNT3, and VNT4, see Table 1 for composition of each) were mixed with N fertilizer at different doses: dose 1 was 83 ml kg N^–1^, dose 2 was 166 ml kg N^–1^, and dose 3 was the equivalent of 3 × 5 L ha^–1^. Dose 3 was tested only with VNT4. Plants were harvested at maturity (GS89). Bars indicate mean ± SE. Different letters denote significant differences according to Fisher’s test (*p* < 0.05; *n* = 3).

VNT4-D3 is the only treatment that significantly increased total plant N compared with the control (+ 15 mg N plant^–1^, Figure 2A). Moreover, a higher NHI was observed for Glutacetine®, VNT1-D1, and VNT4-D3 (+ 12.2, + 11, and + 19.3% compared with the control group, Figure 2B) and a better NUE with VNT4-D3 (Table 2). Remarkably, total grain N was increased only by VNT4-D3 compared with the control plants (+ 24.5 mg N plant^–1^, Figure 2C). As expected, higher grain yields of VNT1, VNT2-D2, VNT3-D1, and VNT4 were also associated with the decline in grain N concentrations (Figure 2D). Regarding the influence of the formulations on N concentration and partitioning in wheat at maturity, a significant reduction in root N concentration was reported for Glutacetine®, VNT1 (doses 1 and 2), VNT3-D1, and VNT4 (doses 2 and 3) (−31.2, −22.6, −30.4, −25, −21.2, and −51.7%, respectively, compared with the control, Figure 2E). Similar results were recorded for N concentration in straws for Glutacetine® and VNT4-D3 alone (1.03% for the control plants versus 0.78% for Glutacetine® and 0.71% DW for VNT4-D3, Figure 2F). In comparison with the control plants, these results suggest that the formulations result in better N remobilization from roots and straw toward the grain.

#### Impact of the Formulations on N Uptake and N Remobilization Between Heading and Maturity

**FIGURE 3:**
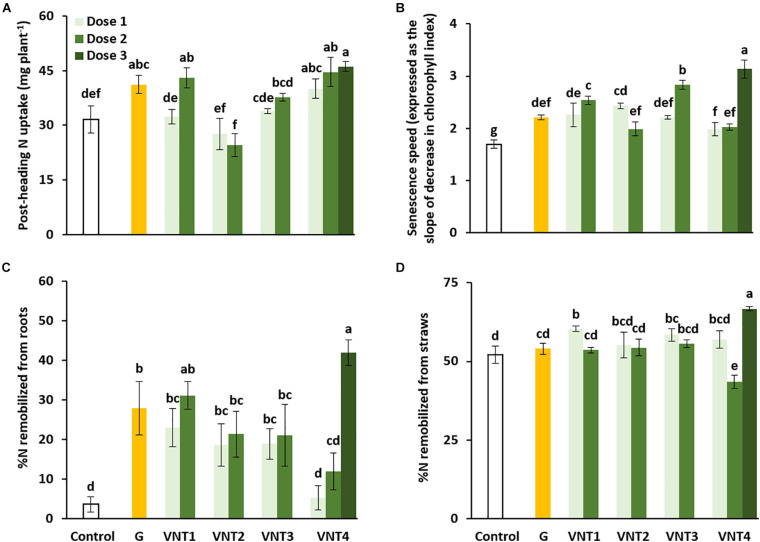
Effect of biostimulant formulations mixed with N fertilizer on post-heading N uptake, senescence speed, and N remobilization efficiency in wheat (*Triticum aestivum* L.). **(A)** Post-heading N uptake, **(B)** senescence speed (expressed as the slope of the decrease in the chlorophyll index, see the section “Materials and Methods” for details), and %N remobilized from roots **(C)** and straw **(D)**. Plant culture was carried out under semi-hydroponic conditions on a sand/perlite (1/1) substrate (see the section “Materials and Methods” for details). N was provided at the tillering (eq. 50 kg N ha^–1^), the 2 nodes (eq. 80 kg N ha^–1^), and the heading stages (eq. 20 kg N ha^–1^). Glutacetine® (G) was mixed with N fertilizer at the heading stage at a dose of 5 L ha^–1^ and applied as a foliar treatment. Four other formulations (VNT1, VNT2, VNT3, and VNT4, see Table 1 for composition of each) were mixed with N fertilizer at different doses: dose 1 was 83 ml kg N^–1^, dose 2 was 166 ml kg N^–1^, and dose 3 was the equivalent of 3 × 5 L ha^–1^. Dose 3 was tested only with VNT4. Plants were harvested at heading (GS59) and maturity (GS89). Bars indicate mean ± SE. Different letters denote significant differences according to Fisher’s test (*p* < 0.05; *n* = 3).

The N amounts observed in the grain at heading and maturity (final harvest) are used to calculate post-heading N uptake and N remobilization efficiency. Total grain N (Figure 2C) was higher than total post-heading N uptake (calculated as the difference between total plant N at maturity and total plant N at heading, Figure 3A), indicating that (i) N uptake after heading was strongly induced by Glutacetine®, VNT1-D2, and VNT4 (Figure 3A) and (ii) there was a net rate of remobilization of N from the vegetative parts toward the grain (Figures 3C,D). Indeed, compared with the control plants, we measured higher uptakes of 9.6 (Glutacetine®), 11.4 (VNT1-D2), 8.4 (VNT4-D1), 13 (VNT4-D2), and 14.5 mg N plant^–1^ (VNT4-D3).

In addition, the senescence speed (estimated as the slope of decline in chlorophyll index between post-flowering and maturity) was measured for each treatment using an optical sensor system (see the Materials and Methods section for more details). Irrespective of the formulation, the slope of the decrease in the chlorophyll index between post-flowering and maturity significantly increased (Figure 3B). To better understand the effect of the formulations tested, we calculated the rate of N remobilized from roots and straw between the heading and maturity stages. Interestingly, all formulations (except VNT4-D1 and D2) increased the rate of N remobilization from roots (Figure 3C) with a major effect from VNT4-D3 (+ 38.3% compared with the control group). VNT1-D1, VNT3-D1, and VNT4-D3 also improved remobilization efficiency in the straw (Figure 3D). Together, these results strongly suggest that VNT4 at dose 3 increased N uptake after heading and accelerated senescence, which improved N remobilization from roots and straw in particular, leading to an increase in yield and NHI.

#### Impact of Biostimulant Formulations on Wheat Grain Quality

##### Changes in the grain ionome

ICP-MS analysis of the grain macronutrient composition revealed that no consistent change was observed for K, and that only VNT4-D2 reduced the %P concentration in the grain (−18.5%, Table 2). VNT4 (doses 2 and 3) also decreased the Mg and Ca concentrations (Table 2). Furthermore, S was significantly lower relative to the control group with all formulations except VNT2-D1 (Table 2). Regarding Na concentrations, only VNT4-D3 maintained the level of control plants, whereas all other formulations significantly decreased %Na (Table 2). Fe was also reduced for all formulations (from 3.3 to 14.6 ppm), but the decline was only significant for VNT1, VNT2-D2, and VNT3 (Table 2). Among the micronutrients, there was no consistent change in Zn concentration in the grain, a crucial nutrient for human health. Compared with the control grain, VNT3-D2 decreased the Mn concentration (−13.4%, Table 2), whereas VNT2-D2 and VNT4-D1 reduced the Cu concentration (−7.3 and −6.8%, respectively, Table 2). In contrast, Mo and B contents were both improved by VNT2-D1 (+ 0.9 and + 0.3 ppm, respectively, Table 2), and Glutacetine® also increased B concentrations in the grain (+ 0.3 ppm, Table 2). Finally, Se was also enhanced by VNT2-D1 and VNT4-D2, and VNT2, VNT3, and VNT4 (only at D2 and D3) increased Cd concentrations in the grain (Table 2).

##### Phytate contents

**FIGURE 4:**
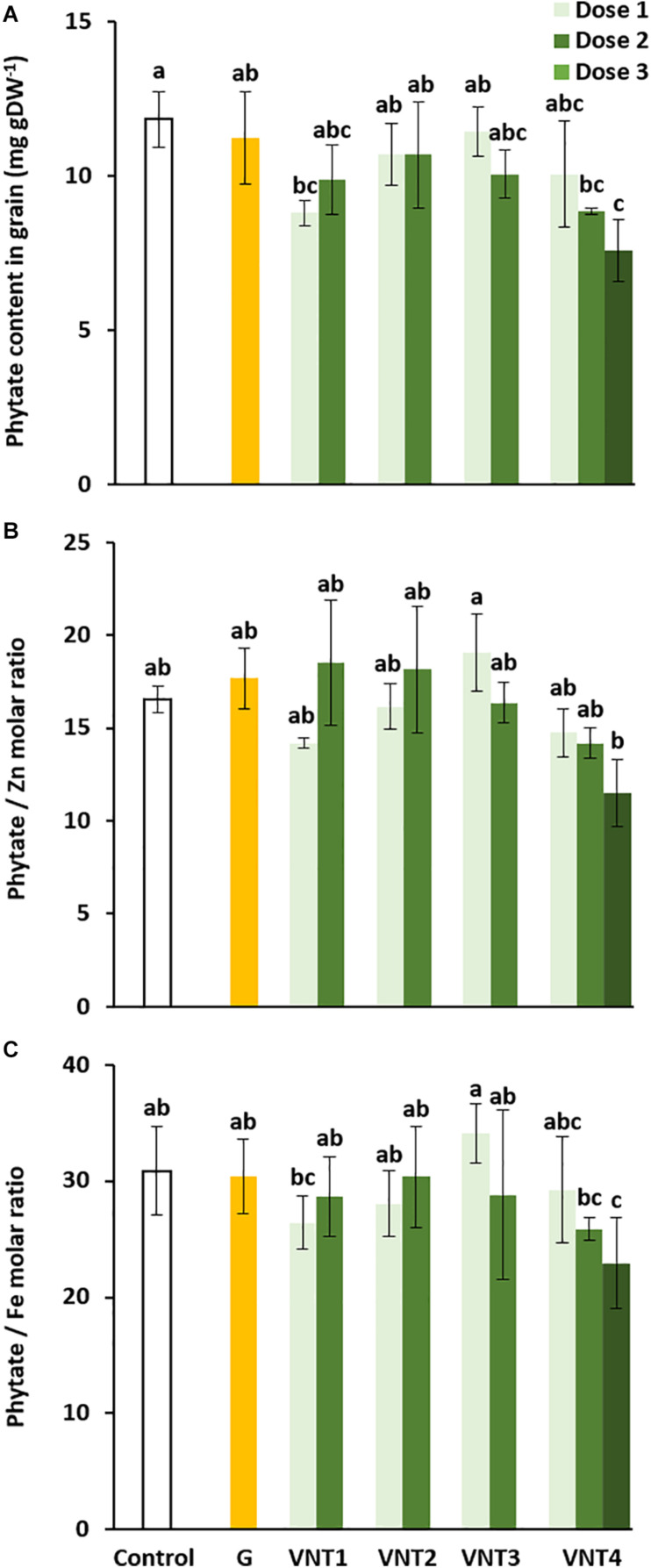
Effect of biostimulant formulations mixed with N fertilizer on phytate, zinc, and iron content in wheat (*Triticum aestivum* L.) grain. **(A)** Phytate content in grain, **(B)** the phytate/Zn molar ratio, and **(C)** the phytate/Fe molar ratio. Plant culture was carried out under semi-hydroponic conditions on a sand/perlite (1/1) substrate (see the section “Materials and Methods” for details). N was provided at the tillering (eq. 50 kg N ha^–1^), the 2 nodes (eq. 80 kg N ha^–1^), and the heading stages (eq. 20 kg N ha^–1^). Glutacetine® (G) was mixed with N fertilizer at the heading stage at a dose of 5 L ha^–1^ and applied as a foliar treatment. Four other formulations (VNT1, VNT2, VNT3, and VNT4, see Table 1 for composition of each) were mixed with N fertilizer at different doses: dose 1 was 83 ml kg N^–1^, dose 2 was 166 ml kg N^–1^, and dose 3 was the equivalent of 3 × 5 L ha^–1^. Dose 3 was tested only with VNT4. Plants were harvested at maturity (GS89). Bars indicate mean ± SE. Different letters denote significant differences according to Fisher’s test (*p* < 0.05; *n* = 3).

As previously observed for P concentrations (Table 2), application of VNT1-D1 and VNT4 (doses 2 and 3) reduced the phytate content (Figure 4A), which has antinutritional properties. The response of wheat plants to the formulations was then investigated with respect to the bioavailability of elements. We thus calculated the phytate/Zn and phytate/Fe molar ratios, which are good indicators of Zn/Fe availability in wheat grain for humans (Brown et al., 2004; Ciccolini et al., 2017). There was no consistent change in Zn bioavailability compared with the control (Figure 4B). However, a significant reduction in the phytate/Fe molar ratio was observed for VNT4-D3 compared with the control group (Figure 4C), indicating that VNT4 at the third dose was able to augment the iron bioavailability in wheat flour.

##### Grain proteome

**FIGURE 5:**
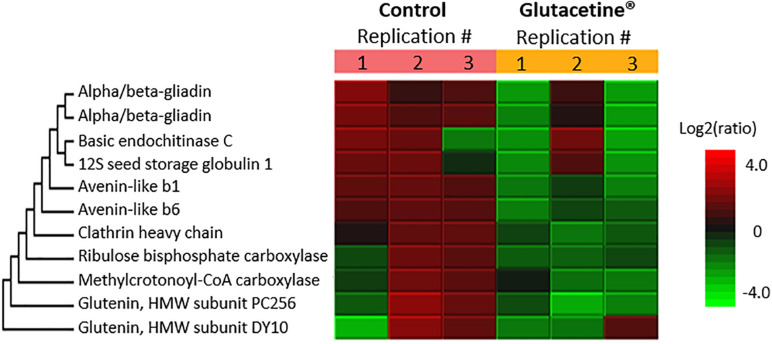
Effect of the Glutacetine® formulation mixed with N fertilizer on the wheat (*Triticum aestivum* L.) grain proteome. Plant culture was carried out under semi-hydroponic conditions on a sand/perlite (1/1) substrate (see the section “Materials and Methods” for details). N was provided at the tillering (eq. 50 kg N ha^–1^), the 2 nodes (eq. 80 kg N ha^–1^), and the heading stages (eq. 20 kg N ha^–1^). Glutacetine® was mixed with N fertilizer at the heading stage at a dose of 5 L ha^–1^ and applied as a foliar treatment. Seeds were harvested at maturity (GS89). A 1.5-fold increase in relative abundance and a Peaks Q threshold ≥ 20 were used to determine enriched peptides/proteins. Only proteins with significant differences in abundance between both treatments are presented.

The response of wheat plants to the formulations was further investigated in terms of quantitative proteomic changes in the flour. Proteomic analysis was focused on the Glutacetine® treatment because it was the only foliar treatment at heading that might have significantly affected the grain quality. Almost 1,900 proteins were detected (1,835 for control grain and 1,842 for grain under Glutacetine® treatment), leading to the identification of 1,850 proteins in total. Even though the protein content was not significantly different compared with the control (Table 2), proteomics revealed strong differences between these treatments for 11 proteins (Figure 5). Indeed, the amount of seed storage proteins was significantly lower under Glutacetine® treatment than under the control plants (Figure 5). Two α/β gliadins, which are strongly linked to celiac disease (Sollid et al., 2012; Shewry and Tatham, 2016), were greatly reduced by Glutacetine® treatment (Figure 5). Gliadins include members with a large repetitive domain and a conserved set of cysteine residues (α/β- and γ-gliadins), members with a repetitive domain but no cysteine (ω-gliadins), and members with low molecular weight gliadins (LMWGs), also known as avenin-like proteins (Zhang et al., 2018). The avenin-like proteins (b1 and b6), which were reduced in the Glutacetine® treatment (Figure 5), are also rich in cysteine (18 or 19 residues), which form intermolecular disulfide bonds and participate in glutenin polymerization (Ma et al., 2013; Wang et al., 2019b). These proteins are known to have impact on dough proprieties (Chen et al., 2016) and antifungal activities (Wang et al., 2019b). Some of these changes could be related to the reduction in S content in grain with Glutacetine® treatment compared with the control group (Table 3) and changed the N-to-S ratio (Supplementary Figure 3, Bonnot et al., 2017; Dai et al., 2015). Foliar application of Glutacetine® also decreased other storage proteins. Regarding globulin storage proteins, Gell et al. (2017) have recently characterized this family of storage proteins in relation to celiac disease. Our results showed that Glutacetine® led to a lower accumulation of 12S seed storage globulin 1 (−55.9% compared with the control, Supplementary Table 4). It is also well documented that the level of fertilizer inputs has a considerable effect on glutenin composition (Yue et al., 2007; Tóth et al., 2019). In our study, two glutenin HMW subunits (PC256 and DY10) were reduced under Glutacetine® treatment; this result is related to the N amount per grain as the amount of glutenin varied in direct proportion (Plessis et al., 2013). A reduction in metabolic proteins (endochitinase, clathrin heavy chain, ribulose bisphosphate carboxylase, and methylcrotonyl-CoA carboxylase) was also observed in response to Glutacetine®. Chitinase activity and clathrin in wheat grain might play roles in responses to infections in plant (Guo et al., 2012; Ekanayake et al., 2019), and methylcrotonyl-CoA carboxylase is an enzyme required for leucine catabolism and may be important during seed development and germination (Ding et al., 2012). These proteomics results highlighted the significant impact of Glutacetine® on wheat grain quality.

**TABLE 3:** Effects of formulations on the composition of macro- and micronutrients in wheat grains (Experiment 1). The relative content (expressed as ppm) of each element is determined by ICP-MS.

	K	P	S	Mg	Ca	Na	Fe
**Control**	4,821.3 ± 168.4ab	2,382.7 ± 47.8ab	1,558.2 ± 56.4a	1,294.3 ± 23.8abc	715.0 ± 35.8a	21.0 ± 3.5a	60.4 ± 3.0a
**Glutacetine®**	4,715.1 ± 113.2ab	2,346.4 ± 84.0ab	1,324.8 ± 14.3bc	1,315.5 ± 74.1ab	673.9 ± 18.3ab	11.5 ± 0.7bcd	53.5 ± 2.2ab
**VNT1**							
Dose 1	4,657.2 ± 190.8ab	2,296.0 ± 207.3ab	1,220.3 ± 112.4cde	1,231.8 ± 68.1bcd	689.0 ± 16.2ab	11.3 ± 1.6bcd	52.5 ± 3.2bc
Dose 2	4,616.6 ± 140.7ab	2,257.0 ± 50.0abc	1,120.2 ± 23.7de	1,210.1 ± 10.0bcd	700.2 ± 43.4ab	12.4 ± 2.5bc	45.8 ± 3.1c
**VNT2**							
Dose 1	4,925.5 ± 360.7a	2,532.2 ± 69.2a	1,447.3 ± 90.8ab	1,387.9 ± 16.7a	693.3 ± 16.8ab	9.1 ± 3.2cd	55.8 ± 3.1ab
Dose 2	4,371.8 ± 49.4ab	2,215.3 ± 82.0bc	1,273.3 ± 125.5bcde	1,202.3 ± 39.6bcd	691.4 ± 31.4ab	6.6 ± 2.4cd	50.1 ± 2.1bc
**VNT3**							
Dose 1	4,465.5 ± 30.2ab	2,185.3 ± 70.7bc	1,244.1 ± 91.7cde	1,251.2 ± 15.5bcd	722.4 ± 53.1a	5.5 ± 2.4d	50.9 ± 2.6bc
Dose 2	4,697.4 ± 543.9ab	2,228.5 ± 204.8bc	1,311.2 ± 134.4bcd	1,204.6 ± 86.0bcd	694.4 ± 48.4ab	12.6 ± 4.6bc	52.0 ± 3.1bc
**VNT4**							
Dose 1	4,304.7 ± 218.1b	2,127.8 ± 126.7bc	1,192.0 ± 75.0cde	1,192.9 ± 37.0cd	650.9 ± 17.4abc	7.3 ± 1.8cd	57.1 ± 5.5ab
Dose 2	4,406.3 ± 81.3ab	2,010.9 ± 106.4c	1,163.1 ± 60.7cde	1,145.4 ± 59.1d	631.5 ± 16.7bc	6.2 ± 1.3d	53.1 ± 2.6abc
Dose 3	4,675.1 ± 198.7ab	2,121.7 ± 78.5bc	1,099.8 ± 2.3e	1,157.5 ± 36.7d	580.7 ± 12.5c	15.6 ± 1.3ab	56.3 ± 2.5ab

	**Zn**	**Mn**	**Cu**	**Mo**	**B**	**Cd**	**Se**

**Control**	38.4 ± 3.2	29.4 ± 1.7ab	7.6 ± 0.11ab	6.1 ± 0.21bcd	2.3 ± 0.11c	0.046 ± 0.0022c	0.0326 ± 0.0071bc
**Glutacetine®**	36.5 ± 0.9	30.2 ± 0.2ab	7.8 ± 0.36a	6.1 ± 0.35bcd	2.6 ± 0.13ab	0.046 ± 0.0022c	0.0326 ± 0.0034bc
**VNT1**							
Dose 1	33.2 ± 1.9	27.0 ± 2.0bc	7.5 ± 0.03abc	5.6 ± 0.13d	2.4 ± 0.09abc	0.045 ± 0.0008c	0.0262 ± 0.0011c
Dose 2	34.1 ± 0.7	28.3 ± 1.2abc	7.3 ± 0.12bcd	5.7 ± 0.36d	2.4 ± 0.05abc	0.045 ± 0.0016c	0.0277 ± 0.0039c
**VNT2**							
Dose 1	37.8 ± 2.1	28.8 ± 0.5abc	7.8 ± 0.28a	7.0 ± 0.16a	2.6 ± 0.07a	0.057 ± 0.0013a	0.0471 ± 0.0050a
Dose 2	34.8 ± 2.1	29.1 ± 2.0ab	6.8 ± 0.15d	6.5 ± 0.29abc	2.3 ± 0.07c	0.052 ± 0.0011b	0.0435 ± 0.0036ab
**VNT3**							
Dose 1	33.3 ± 2.1	27.9 ± 0.5abc	7.3 ± 0.24abc	5.9 ± 0.44cd	2.4 ± 0.15bc	0.052 ± 0.0011b	0.0424 ± 0.0012ab
Dose 2	37.0 ± 7.8	25.4 ± 0.4c	7.2 ± 0.09bcd	6.7 ± 0.39ab	2.4 ± 0.05bc	0.055 ± 0.0023ab	0.0410 ± 0.0026ab
**VNT4**							
Dose 1	34.0 ± 2.8	28.3 ± 2.7abc	7.1 ± 0.23cd	6.3 ± 0.25abcd	2.4 ± 0.11abc	0.047 ± 0.0034c	0.0352 ± 0.0107abc
Dose 2	34.0 ± 1.7	27.1 ± 0.6bc	7.2 ± 0.11bcd	6.0 ± 0.43bcd	2.3 ± 0.06bc	0.052 ± 0.0005b	0.0453 ± 0.0041a
Dose 3	33.1 ± 2.0	31.3 ± 1.2a	7.2 ± 0.15bcd	6.1 ± 0.42bcd	2.2 ± 0.05c	0.054 ± 0.0016ab	0.0456 ± 0.0045ab

*Values indicate mean ± SE. Different letters denote significant differences according to Fisher’s test (*p* < 0.05; *n* = 3).*

### Impact of VNT4 Under Contrasting Field Conditions (Exp. 2)

**FIGURE 6:**
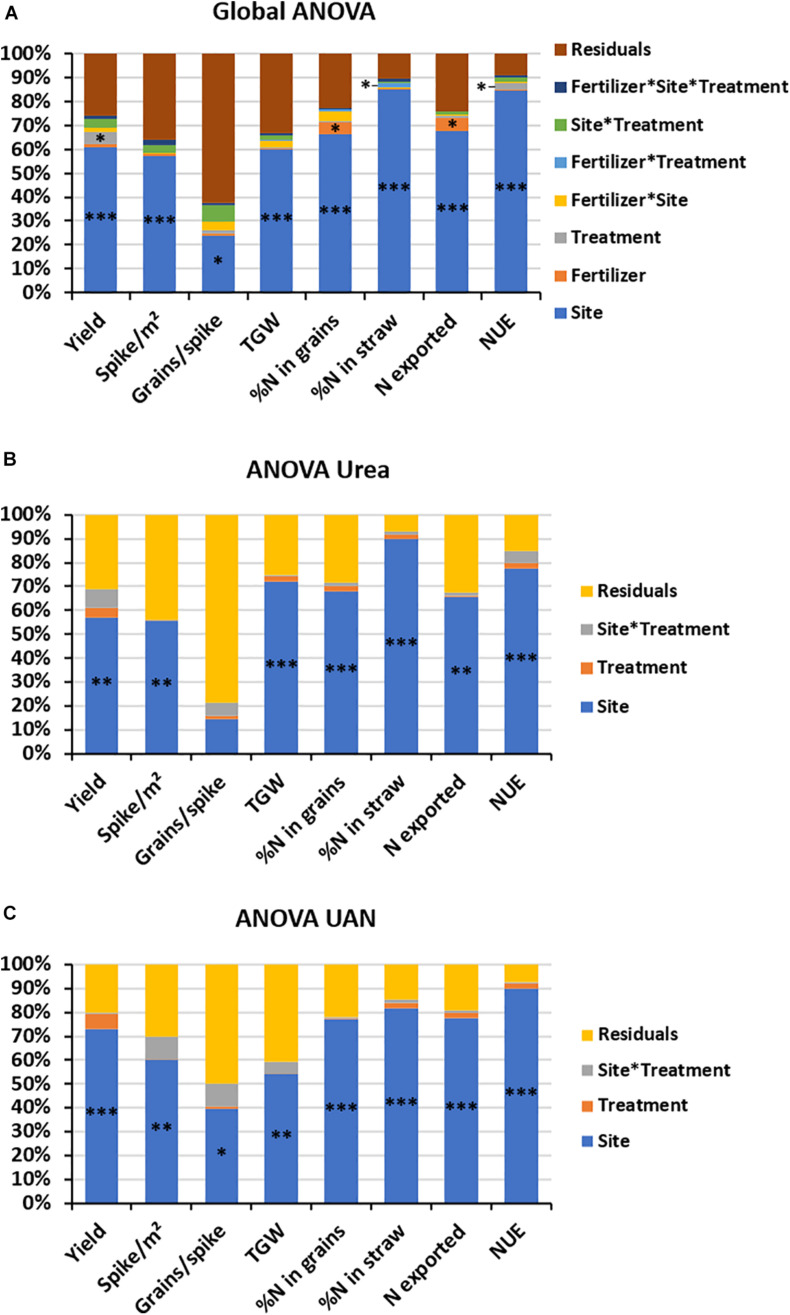
Schematic representation of ANOVA of yield (t ha^–1^), spike number per square meter, grain number per spike, thousand grain weight (TGW), N content in grains (%), N content in straw (%), N exported by grains (kg ha^–1^), and NUE (kg kg^–1^). **(A)** Global ANOVA using data obtained in the three sites and with the two fertilizers, **(B)** ANOVA urea using data obtained in the three sites with urea fertilization, and **(C)** ANOVA UAN using data obtained in the three sites with UAN fertilization. ^∗∗∗^*p* < 0.001, ^∗∗^*p* < 0.01, and ^∗^*p* < 0.05.

To validate the effect of VNT4, three field trials were carried out in contrasting sites in Normandy (France, Supplementary Table 1). Global ANOVA revealed a strong site effect for all parameters analyzed (Figure 6A). This analysis also indicated an effect of fertilizer type on N content in grain and on N exported by grains (Figure 6A). Interestingly, VNT4 influenced yield and NUE (Figure 6A). Indeed, VNT4 treatment improved these parameters irrespective of the site and the fertilizer used except with urea in site 1 (Table 4). There is also a significant interaction between fertilizer used and treatment on N content in straw (Figure 6A). In fact, mixed with urea, VNT4 tended to decrease N concentration in straw, whereas it tended to increase it when mixed with UAN (Table 4). Moreover, the ANOVA of urea fertilization showed only a site effect for all parameter except grain number per spike (Figure 6B). However, the ANOVA of UAN indicated a site effect on grain number per spike (Figure 6C). In addition, there is a slight effect of VNT4 on yield and NUE when mixed with UAN (*p* = 0.073 and *p* = 0.075, respectively, Figure 6C). Finally, on field trial 1 (site 1), N content was influenced by fertilizer type, whereas no consistent change was observed on site 2 (Supplementary Figure 4). Interestingly, VNT4 improved yield and NUE on site 3 with a slight increase of N exported by grains (*p* = 0.083), whereas fertilizer type only influenced N exported by grains (Supplementary Figure 4). Regarding yield, VNT4 mixed with urea increased it in sites 2 and 3 but decreased it in site 1, whereas when VNT4 is mixed with UAN, yield increased irrespective of the site (from + 246 to 574 kg ha^–1^, with a mean of 463 kg ha^–1^, Table 4). This improvement is due either to the increase of spike number per square meter (sites 1 and 3) or to the better grain number per spike (site 2, Table 4). On N partitioning, there was no consistent change involved by VNT4 in straw and grain. But, except in site 1 with urea, VNT4 increased N exported by grains and NUE (Table 4). Indeed, when mixed with UAN, VNT4 increased N exported by 12 kg N ha^–1^ and NUE by 4%. These results under contrasting field conditions confirmed our previous results under controlled conditions on agronomic parameters especially when VNT4 is mixed with UAN.

**TABLE 4:** Effects of VNT4 on yield, spike number per square meter, TGW, N content in grains and straw, N exported by grains, and NUE under contrasting field conditions (Experiment 2).

	Yield (t ha^–^^1^)	Spike m^–^^2^	Grains spike^–^^1^	TGW (g)	%N in grains	%N in straw	N exported (kg ha^–^^1^)	NUE (kg kg^–^^1^)
**Site 1**								
**Urea**								
Control	12.05 ± 0.16bc	576.0 ± 18.5ab	42.87 ± 1.23abc	54.13 ± 0.52a	1.88 ± 0.02de	0.70 ± 0.00ab	226.9 ± 2.95bc	79.3 ± 1.03ab
VNT4	11.64 ± 0.27bcd	576.0 ± 27.7ab	40.96 ± 1.94abc	54.79 ± 0.78a	1.88 ± 0.02de	0.71 ± 0.02ab	219.3 ± 5.86c	76.6 ± 1.78ab
**UAN**								
Control	11.84 ± 0.14bc	541.3 ± 8.2abcd	45.28 ± 1.36ab	53.40 ± 0.57ab	1.70 ± 0.07e	0.64 ± 0.02b	201.5 ± 9.14c	77.9 ± 0.92ab
VNT4	12.08 ± 0.12bc	588.0 ± 18.5a	44.36 ± 1.82abc	51.56 ± 1.24abc	1.68 ± 0.04e	0.66 ± 0.03ab	202.8 ± 7.44c	79.5 ± 0.82a
**Site 2**								
**Urea**								
Control	12.32 ± 0.26ab	600.0 ± 23.1a	39.30 ± 1.62bc	53.85 ± 0.95a	2.25 ± 0.06a	0.76 ± 0.04a	277.8 ± 13.21a	58.7 ± 1.25fg
VNT4	13.25 ± 0.67a	598.7 ± 23.6a	42.95 ± 3.78abc	54.71 ± 1.04a	2.17 ± 0.06abc	0.67 ± 0.01ab	288.2 ± 20.07a	63.1 ± 3.18ef
**UAN**								
Control	11.95 ± 0.31bc	625.3 ± 14.9a	36.46 ± 0.97c	54.59 ± 1.65a	2.21 ± 0.01ab	0.66 ± 0.03ab	264.7 ± 8.26ab	56.9 ± 1.46g
VNT4	12.52 ± 0.19ab	566.7 ± 19.5abc	42.23 ± 1.74abc	54.82 ± 0.73a	2.24 ± 0.11a	0.76 ± 0.06a	280.2 ± 11.49a	59.6 ± 0.88fg
**Site 3**								
**Urea**								
Control	10.37 ± 0.08e	486.0 ± 16.2cde	44.70 ± 1.40abc	47.94 ± 0.21c	2.08 ± 0.06abcd	0.39 ± 0.01c	215.9 ± 4.14c	68.2 ± 0.55cde
VNT4	11.20 ± 0.07cde	494.7 ± 12.6bcde	46.08 ± 2.14ab	49.35 ± 1.09c	2.02 ± 0.03bcd	0.33 ± 0.03c	225.9 ± 3.82c	73.7 ± 0.48bc
**UAN**								
Control	10.18 ± 0.26e	454.0 ± 37.7e	47.82 ± 3.42a	47.84 ± 0.26c	1.88 ± 0.04de	0.37 ± 0.01c	191.9 ± 9.19c	66.9 ± 1.73de
VNT4	10.75 ± 0.20de	475.3 ± 10.3de	46.58 ± 0.48ab	49.56 ± 1.29bc	1.97 ± 0.05cd	0.38 ± 0.02c	211.1 ± 2.53c	70.7 ± 1.29cd

*Values indicate mean ± SE. Different letters denote significant differences according to Fisher’s test (*p* < 0.05; *n* = 3).*

## Discussion

The goals of this study were to provide a detailed analysis of the effects of N fertilizers containing diverse formulations of Glutacetine®-based biostimulants on physiological traits, N uptake and partitioning, agronomic traits, and different criteria related to wheat grain quality. VNT formulations were mixed with N fertilizers and applied during each N supply, whereas Glutacetine® was mixed with N fertilizers and sprayed during the last supply alone. Clearly, the present work does not provide any functional dissection of N metabolism in the plants but aims to highlight the impacts of the formulations during senescence and on N use efficiency in wheat plants grown under realistic conditions in terms of plant size, canopy structure, and grain sink strength.

### Formulations Enhanced Straw Biomass, Increased Grain Yields, and Impacted the Components of Grain Yield

Winter wheat cv. Récital achieves an average grain yield of 2.84 g plant^–1^ under field conditions (equivalent at 711 g m^–2^, Bogard et al., 2010). Under semi-hydroponic conditions, Récital attains 2.8–7.8 g plant^–1^, depending on the N conditions (Taulemesse et al., 2015). In the present work, the grain yield of Récital was between 2.8 and 4.3 g plant^–1^ (Figure 1B), indicating that our experimental design led to grain yields that were very close to those obtained in field trials. Values obtained for TGW and grain density were low compared with field trials, which have been reported above 40 g for TGW (Popko et al., 2018) and above 75 kg hl^–1^ in terms of density (Manley et al., 2009). However, Baillot et al. (2018) showed a reduction in the TGW between field and greenhouse conditions. These trends are in accordance with our values (27–33 g).

In our study, several biostimulants (VNT1, VNT3-D1, VNT4-D2, and D3) resulted in a significant increase in grain yield (up to + 51.7% for VNT4-D3 compared with control, Figure 1B). This improvement is due in particular to the increase in the grain number per spike (Figure 1E) and the fruiting efficiency (Figure 1D). In contrast, Glutacetine®-based formulations did not show consistent changes in tiller number and spike number per plant (Table 1). In accordance with our findings, Popko et al. (2018) also observed a positive effect of amino acid-based biostimulants used in foliar treatments on plant growth, grain yield, and ear number per square meter in winter wheat. It is also well documented that other biostimulants, such as arbuscular mycorrhizal fungi in association with crops, have positive impacts on vegetative and grain DW (Bücking and Kafle, 2015; Criado et al., 2015; Verzeaux et al., 2017). When used in soil or in foliar treatments, Subbarao et al. (2015) showed that protein hydrolysate enhanced plant growth (roots and shoots) and seed yield in rice, finger millet, radish, and cowpea. Moreover, soil application was more effective than foliar application, which is in accordance with our findings. Similar results were obtained by Nguyen et al. (2019), who tested the effect of *Bacillus* strains on wheat under contrasting N supplies.

Our results also showed that straw biomass at the maturity stage correlated with grain yield (*r*^2^ = 0.65). This correlation suggests that biostimulant treatments leading to high above-ground biomass produced higher grain yield, as previously reported when N nutrition is not limiting (Gaju et al., 2016). This is a consequence of physiological mechanisms that contribute to high straw biomass production and that may result in enhanced N storage capacity in the plant. In our findings, when VNT4 was applied at 5 L ha^–1^ with each N supply (D3), the capacity of N storage was significantly increased but only after heading. Indeed, VNT4-D3 did not change the N amount at heading compared with control (Supplementary Figure 2), but the N amount was improved during the post-heading period (Figure 2E). This is in agreement with data from the literature that describe biostimulants as acting by increasing plant mineral uptake and by improving nutrient use efficiency (Calvo et al., 2014; du Jardin, 2015). In fact, different kinds of biostimulants (protein hydrolysate, plant extracts, *Trichoderma*) on various plant species, such as tomato (*Solanum lycopersicum* L.), dwarf pea (*Pisum sativum* L.), corn (*Zea mays* L.), lettuce (*Lactuca sativa* L.), and rocket (*Eruca sativa* Mill.), are able to improve NUE (Colla et al., 2014; Fiorentino et al., 2018; Sestili et al., 2018; Di Mola et al., 2019).

### Formulations Induced Differences in Post-heading N Uptake and N Remobilization

As previously reported in durum wheat (Pichereaux et al., 2019), we observed deviations from the grain N concentration to grain yield negative correlation for VNT1-D2, VNT2 (doses 1 and 2), and VNT4 (doses 1 and 3). Bogard et al. (2010) correlated this deviation to post-anthesis N uptake in winter wheat, which explains the results of VNT1-D2 and VNT4 (Figure 3A). Root activity was maintained under these treatments during the grain-filling period (Figure 3A). Therefore, VNT1-D2 and VNT4-D3 probably maintain post-flowering N uptake during grain development, and this then guaranteed that the protein content was adequate for high grain yield. Indeed, Taulemesse et al. (2016) demonstrated that the grain N concentration at maturity and N uptake from flowering to GS65 + 250 degree days were correlated (*r*^2^ = 0.8). The post-heading N uptake of VNT4 at dose 2 allowed the wheat plants to increase yield alone because their protein content correlated with this seed yield (*r*^2^ = 0.96). However, it has also been observed that primary N assimilation decreases with senescence, whereas N recycling and remobilization enzymes are stimulated (Masclaux-Daubresse et al., 2010). In our study, the formulations seemed to maintain N uptake and assimilation during the senescence process.

Three main hypotheses can be proposed to explain the physiological mechanisms underlying the variability in the formulation responses during post-heading N uptake for a given total N at heading. The first postulate is related to phenotypic differences in accessing soil N. In our study, VNT4-D2 had a significantly higher root DW than VNT1-D2, which could partially explain the different behaviors between these two treatments. Indeed, VNT4 at the second dose was associated with a slow senescence speed compared with VNT1-D2 (Figure 3B) but had a better rate of N remobilization from roots (Figure 3C) and straws (Figure 3D).

The second hypothesis is related to the interactions of biostimulants on the regulation of N uptake by the plant N status. It is well documented that plant N status regulates N influx (Barneix, 2007), and in particular regulates the transcription of nitrate transporters with the phloem amino acid concentration (Glass, 2002). Amino acids contained in our biostimulant formulations may have interfered with and altered the internal plant signaling associated with plant N status, and this might explain the differences in post-heading N uptake and remobilization.

The third hypothesis to explain the variability in the responses of post-heading N uptake may be energy related. Delaying senescence is able to prolong photosynthetic activity (Havé et al., 2017) and therefore maintain the delivery of carbon to the roots in order to maintain a higher capacity to take up N from the substrate during the post-heading period. Regarding our N index data, a value below 2.5 indicates that the plant is in senescence (with a general yellowing of leaves). Therefore, senescence began 2 days before the control group following the VNT4 third dose, whereas senescence was delayed for 4 days under VNT1-D2 (Supplementary Figure 5). Delaying leaf senescence is also associated with increased seed yield or grain protein content, depending on the environments considered (Bogard et al., 2011). This differential effect on grain yield and grain protein concentration of delaying leaf senescence is related to N availability during the post-anthesis period. In our findings, only VNT1-D2 and VNT4-D2 were associated with delayed senescence and increased N uptake after heading.

These three hypotheses are not mutually exclusive; data are also currently lacking in terms of rejecting one or the other. It is possible that the physiological differences observed for post-anthesis N uptake at a given total N at anthesis are caused by the three proposed processes and their potential interactions.

Furthermore, our study showed that the formulations strongly increased remobilization efficiency from roots and/or the straw toward the grain. This could be due to a higher proteolysis and autophagic activity that improved N remobilization efficiency and grain filling (Su et al., 2020). Regarding remobilization, plant extracts might reprogram N distribution and remobilize it from amino acids, especially glutamate, under lower nutrient conditions (Carillo et al., 2019), and biostimulants, such silicon, could have a stay green effect (Haddad et al., 2018).

Altogether, these results demonstrated that our biostimulants mixed with N fertilizer have strong impacts on N allocation due to a major effect after the heading stage on N uptake and N remobilization efficiency, which leads to improve fruiting efficiency and grain number per spike to finally increase seed yield.

### Biostimulants Supplied to N Fertilizer Changed the Ionome and Phytate Concentrations and Affected Protein Concentration and Quality in Wheat Grain

#### Grain Ionome and Phytate Concentrations

Minerals are essential in human nutrition. Fe, Ca, Zn, and Cl are the most in need of fortification in cereals in order to reduce nutrition deficiency across the world (Gharibzahedi and Jafari, 2017). In our experimental design, macronutrient concentrations in the grain of control plants were in accordance with those found in the literature (Wieser et al., 2004; Rose et al., 2015; Khokhar et al., 2018). The reduction in P by VNT4-D2 could enhance sustainable management of this element in agricultural production *via* the development of “low seed total P” (Raboy, 2020). Regarding S concentration values, these ranged from 0.11 to 0.16%, which are close to the values obtained by Wieser et al. (2004), who studied the influence of S nutrition in wheat flour quality. Nevertheless, these authors demonstrated that the amounts and proportions of single protein types were strongly affected by different S fertilizer applications, mainly due to a dependence on the Cys and Met contents of each protein type. We propose that the S reduction in grain due to the formulations might have the same consequence. In addition, the N/S ratio, which was impacted by VNT4-D3 (Supplementary Figure 3), is known to modulate the duration of accumulation of S-rich grain storage protein and the rate of accumulation of S-poor grain storage protein (Dai et al., 2015).

In terms of other microelements, we obtained similar results to field conditions for Se and Cu, but higher Mo and B concentrations (Rose et al., 2015; Khokhar et al., 2018; Popko et al., 2018). For Cd, grain concentrations ranged from 0.045 to 0.057 ppm and were thus similar to those measured in hydroponic conditions in durum wheat lines (Perrier et al., 2016; Yan et al., 2018). Although some treatments increased the Cd accumulation in grain, all Cd contents observed in our study were below the maximum grain concentration of 0.2 μg Cd g^–1^ DM that is authorized in Europe (Commission Regulation, 2006).

Wheat is an important dietary source of Fe and Zn for the population, and there is scope to increase grain Fe and Zn concentrations in wheat through breeding or biofortification to help alleviate dietary Fe and Zn deficiencies. In the present study, we found equivalent or higher Fe and Zn contents than under field conditions (Krochmal-Marczak and Sawicka, 2008; Zhao et al., 2009; Rose et al., 2015; Khokhar et al., 2018; Singh et al., 2018). For example, a study that investigated the variation in mineral micronutrient concentrations in grain from wheat lines of diverse origin indicated Fe concentrations that ranged from 28.8 to 50.8 ppm (Zhao et al., 2009), whereas our findings revealed contents ranging from 45.8 to 60.4 ppm. Khokhar et al. (2018) showed that the grain Zn concentration of 36 wheat genotypes varied from 26 to 32 mg kg^–1^. We observed a similar situation with Zn levels, which ranged from 33.1 to 38.4 ppm in our study. Our results are consistent with Zhao et al. (2009) who reported a spread of grain Zn concentrations from 13.5 to 34.5 mg kg^–1^ in 150 field-grown bread wheat lines. A global summary of grain Zn concentrations in field grown wheat recently reported grain Zn concentrations from 20 to 31 mg kg^–1^, which is substantially lower than the biofortification target of 40 mg kg^–1^ for the human diet (Chen et al., 2017). Regarding the strong increases of seed yields (from 11 to 52%), our findings showed slight reduction in Fe (−5 to −17%) and Zn (−1 to −14%) contents. In contrast, it has been recently demonstrated that biostimulants enhance Zn concentration and bioavailability in wheat grain (Wang et al., 2020; Yadav et al., 2020). Nevertheless, in our study, the amount of Fe and Zn exported was significantly higher for VNT4 at dose 3 than for the control group (data not shown), which might be due to better remobilization during monocarpic senescence. In addition, all treatments except VNT2-D2 enhanced the export of these two elements (data not shown).

One of the main impacts on grain quality observed in the present study was the significant reduction in phytate in response to VNT1-D1 and VNT4-D2 and D3 (Figure 4A). Substantial effort has been invested in (i) wheat cultivars with low phytic acid content in the grain (Guttieri et al., 2006; Ficco et al., 2009; Guo et al., 2015; Raboy, 2020) and (ii) biofortification to enhance Fe and Zn contents and availability in wheat grain (Hussain et al., 2012; Ramzani et al., 2016; Gomez-Coronado et al., 2017; Xia et al., 2018). A major contributor to Fe and Zn deficiencies in the human body is the abundance of phytates in food (Gharibzahedi and Jafari, 2017; Raboy, 2020), which are involved in chelating minerals, such as Zn and Fe (Xue et al., 2015). Indeed, phytate to Fe or Zn molar ratios are considered the best indicators of Fe and Zn availability for humans (Brown et al., 2004; Ciccolini et al., 2017). A phytate/Zn molar ratio lower than 15 is equal to high Zn bioavailability (Gargari et al., 2007), whereas a phytate/Fe molar ratio < 1 is indicative of good Fe bioavailability (Hurrell and Egli, 2010). In our study, the phytate/Zn molar ratio was reduced by 15, 16, and 25% for VNT1-D1, VNT4-D2, and VNT4-D3, respectively, but these values were not significant (Figure 4B). We observed the same trend for the phytate/Fe molar ratio (−14, −14, and −30%, respectively), and it was significantly lower for VNT4 at dose 3 (Figure 4C). Therefore, the bioavailability of Fe and Zn in wholemeal flour was improved by VNT4 at the third dose compared with the control. These results are similar to studies that have evaluated the effect of foliar Zn/Fe spraying on quality in wheat grain (Hussain et al., 2012; Xia et al., 2018). However, to the best of our knowledge, this is the first report demonstrating the impact of biostimulants mixed with N fertilizers on the final phytate concentrations in grain.

#### Protein Content and Quality

Regarding protein content, Bogard et al. (2010) reported a mean of 11.25% for Récital, whereas Taulemesse et al. (2015) observed a range from 14.2 to 15.8%; our findings were between the two (from 12.5 to 14.2%), which confirmed that our system was comparable to agronomic practices. It is also well documented that higher yield decreases the N and protein contents in grain (Hawkesford et al., 2018). Accordingly, in the present study, the protein content in wheat grain was highest in the control, whereas all of the formulations reduced it to 12.5% (Table 1). Similar to our results, other biostimulants obtained from marine, fungal, or protein hydrolysates have not been shown to change the protein content in wheat (Popko et al., 2018; Pichereaux et al., 2019). However, none of the treatments from our work resulted in a protein content below the level required by the industry (11.5%) even though this is not the best criterion to ensure baking quality (Rossmann et al., 2019).

We finally investigated the effect of Glutacetine® on the quality of wheat grain proteins using proteomic analysis. In order to identify the largest number of bread wheat proteins, we used a large-scale label-free quantitative proteomics approach based on the analysis of the total protein extracted from flour samples. Foliar application of Glutacetine® with N fertilizer at heading did not significantly affect the grain N concentration (Figure 2) but impacted the quantities of 11 proteins relative to the control (Figure 5). As suggested by Rossmann et al. (2019), these changes in the grain proteome could have been due to the foliar application of N fertilizers at anthesis and might enhance baking quality. Indeed, the same authors showed that foliar N application increased HMW glutenin while decreasing LMW glutenin and α-/β-/γ-gliadins. In contrast, our results showed that Glutacetine® reduced HMW glutenin, α-/β-gliadins, and thus the HMW-GS/LMW-GS ratio, but not the gliadin/HMW-GS ratio. Compared with glutenins, the amount of gliadins was affected disproportionally by the N amount per grain (Supplementary Figure 3) as detailed by Plessis et al. (2013). Glutacetine® also decreased two avenin-like proteins, which are implicated in dough quality (Zhang et al., 2018). Therefore, it is difficult to conclude whether Glutacetine® application led to a significant improvement in the baking quality. However, it is well documented that α-gliadins (Zörb et al., 2013) and 12S seed storage globulins (Gell et al., 2017) are initiators of the inflammatory response in celiac disease patients. In durum wheat, Pichereaux et al. (2019) have reported the effects of marine and fungal biostimulant treatments on the grain proteome, and especially proteins involved in grain technological properties, such as the gluten protein gamma-gliadin, which increased 100-fold following treatment. In contrast, our study suggests that application of Glutacetine® might improve grain quality through a decline in the immunogenic potential of wheat flour (Altenbach et al., 2020). Most of the proteins that trigger immunological reactions are rich in S-amino acids; their decline is thus strongly linked to the reduction in S content in the grain induced by Glutacetine® (Table 3). Therefore, foliar application of N fertilizer coupled with Glutacetine® slightly affected the N/S ratio (Supplementary Figure 3), modulating the accumulation of S-rich and S-poor storage proteins in grain (Dai et al., 2015), and this could have a positive impact on the nutritional and technological qualities of the flour.

### Organic Acids Enhanced the Effect of Glutamic Acid in Wheat Plants

Regarding the composition of formulations, the biggest differences between them were the glutamic acid/organic acids ratio with a minimum of 0.48 for VNT4 and a maximum of 19.6 for VNT1 (Table 1). This important difference highlighted the beneficial impacts of organic acids on the efficiency of glutamic acid in wheat. Indeed, it is well documented that application of glutamic acid improves plant development (Sun and Hong, 2010), especially by enhancing N metabolism (Haghighi, 2012). Glutamic acid could improve photosynthesis due to a better chlorophyll content and velocity of Rubisco; it also induces the activities of nitrate reductase and glutamine synthase, leading to a higher soluble protein content, leaf N content, and N accumulation in leaves (Yu et al., 2010; Haghighi and Silva, 2013). In addition, glutamic acid alleviates abiotic stress, such as cold during the early vegetative stage in rice (Jia et al., 2017). Amino acids as biostimulants also serve as hormone precursors, signaling factors and regulators of N uptake, root development, and antioxidant metabolism (Khan et al., 2019). Better root development supported by the addition of amino acids can enhance root surface for nutrient uptake. In our study, we did not show consistent change in root surface, but there was a strong N uptake and remobilization during the post-heading period compared with the control leading to higher yield and NUE, probably partly due to glutamic acid present in the formulations. In general, glutamic acid is mixed with other amino acids before to be applied on plant (Haghighi et al., 2020; Tsouvaltzis et al., 2020). In the present study, glutamic acid was mixed with organic acids except for VNT1. Interestingly, even if when the dose of glutamic acid was decreased by more than 80% in VNT4 compared with VNT1 (Table 1), the addition of a small amount of organic acids maintained high yield and NUE (Figure 1B and Table 2). VNT4-D3 achieved even better results than VNT1. Thus, mixed glutamic acid with organic acids is efficient to enhance the effects of glutamic acid.

### VNT4 Increased Yield and NUE Under Field Conditions

To verify the positive effects of VNT4 observed under controlled conditions, we then tested VNT4 at dose 3 in three contrasted field trials on three different cultivars of bread wheat. While yields are staging in Europe (Brisson et al., 2010), our results indicated that VNT4 improved agronomic parameters, especially yield and NUE (Figure 6A). VNT4 was more efficient with UAN than urea (Figure 6C and Table 4). Even if there was less significant yield (+ 51.7% yield under controlled conditions, + 4.1% under field conditions), our results under field conditions are in accordance with those obtained under greenhouse conditions: VNT4 enhanced yield and NUE, whereas there is a strong trend to export more N in grains. Nevertheless, the improvement of yield was mainly due to the higher fruiting efficiency and grain number per spike under controlled conditions. In field trials, VNT4 did not change grain number per spike in sites 1 and 3 but improved this parameter in site 2. Indeed, the better yields in sites 1 and 3 were mainly due to the improvement of the spike number per square meter. This difference may be due to the stage of application; the first VNT4 supply was at the end of tillering (BBCH 29) under greenhouse conditions, whereas it was at the beginning of tillering (BBCH 21) under field conditions, which may increase tiller number and thus spike number per square meter. Other biostimulants based on amino acids have also positive effects on spike number per square meter and yield of wheat under field conditions (Popko et al., 2018). Moreover, in this study, the authors did not show consistent change regarding TGW and protein content, which is in agreement with our findings. Indeed, regarding seed quality, while VNT4 decreased N content under greenhouse conditions mainly due to strong yield increase, there was no consistent change under field conditions. Besides, TGW was not affected by VNT4 in both experiments. In the same way, under contrasting N fertilization field trials, significant impacts on wheat grain yield and spike number per square meter were observed with *Bacillus* strains (Nguyen et al., 2019). In rice, controlled-release fertilizer has similar effect on yield and NUE compared with our findings (Sun et al., 2020). In addition, protein hydrolysates are known to have biostimulant properties under field conditions, such as in tomatoes (Polo and Mata, 2017). Finally, other kinds of biostimulants, such as silicon, may enhance yield and seed quality under field conditions, but only under high N fertilization (Laîné et al., 2019). Altogether, these results demonstrated that VNT4 mixed with N fertilizer has a significant impact on yield and NUE, especially when mixed with UAN, which confirmed the results obtained under greenhouse conditions.

## Conclusion

We demonstrated that biostimulants incorporated into N fertilizers may enhance straw DW (VNT1, VNT2-D1, VNT3, and VNT4 at doses 2 and 3) and seed yield (VNT1, VNT3-D1, and VNT4 at the second and third doses) due to an improvement in the fruiting efficiency and the resulting increased number of grains per ear (Glutacetine®, VNT1, VNT2-D1, VNT3, VNT4-D2, and VNT4-D3). These new formulations of biostimulants were also able to enhance the total N in grain (VNT4-D3) and the NHI (Glutacetine®, VNT1-D1, and VNT4-D3). This was due to efficient post-heading N uptake (VNT1-D2 and VNT4 at doses 2 and 3), the acceleration of senescence (all formulations), and a strong N remobilization from roots and straw (VNT4-D3). Further studies with lower doses of N will be undertaken in order to understand the regulation of N assimilation and remobilization enzymes and transporters during senescence because strong modifications during this period have been demonstrated (Havé et al., 2017). Indeed, nitrate reductase (NR) activity is highly correlated with the N absorbed post-flowering and also with grain protein content. Further, glutamine synthetase (GS) activity is even more highly correlated with the amount of N remobilized and grain yield than NR activity, so analysis of these enzymes seems essential (Kichey et al., 2007). The formulations also impacted grain quality by reducing the N concentration in the grain, but the protein content stayed above the 11.5 required by the industry. In an original way, our proteomic analyses highlighted a novel effect of Glutacetine® on gluten relative to the control. Regarding the grain ionome, the formulations mainly affected S, Na, Fe, and Cd contents and the availability of Fe. Altogether, the results demonstrated that winter wheat was positively impacted by VNT1 and VNT4 (mainly at doses 2 and 3), especially for grain yield, agronomic traits, and grain quality. We also showed that mix glutamic acid with organic acids is efficient to enhance the effects of glutamic acid only (VNT4-D3 compared with VNT1). Finally, the field experiments highlighted that VNT4 applied at dose 3 and mixed with urea or UAN significantly increased yield and NUE irrespective of the site.

Sustainable agriculture requires not only effective mineral fertilizers containing macro- and microelements but also biostimulants that are a rich source of biologically active compounds. When mixed with mineral fertilizers, such preparations improve the efficiency of nutrient uptake. Indeed, the new formulations presented in the current work allow significant increases in seed yield and grain quality to be achieved. Greenhouse experiments and field trials will continue to verify these biostimulants according to established principles (Ricci et al., 2019) and enable registration of new biostimulant products for release onto the market.

## Data Availability Statement

The datasets presented in this study can be found in online repositories. The names of the repository/repositories and accession number(s) can be found in the article/Supplementary Material.

## Author Contributions

VM, J-CA, and PG designed the experiments. VM and J-CA conducted all the experiments, analyzed the data, and wrote the manuscript. BB performed the proteomic analysis. All authors contributed to the article and approved the submitted version.

## Conflict of Interest

VM and PG were employed by the company Via Végétale. The remaining authors declare that the research was conducted in the absence of any commercial or financial relationships that could be construed as a potential conflict of interest.
