# Supplementary material for: Biostimulant Effects of Glutacetine® and Its Derived Formulations Mixed With N Fertilizer on Post-heading N Uptake and Remobilization, Seed Yield, and Grain Quality in Winter Wheat
Source: Front Plant Sci. 2020 Nov 13;11:607615. doi: 10.3389/fpls.2020.607615 (PMC7691253; doi:10.3389/fpls.2020.607615)
Supplement: Supplementary file 1 [file Table_1.pdf]

Supplementary Table 1. Location, wheat cultivars and soil properties of the 3 field trials (Exp. 2)

	**Site 1**	**Site 2**	**Site 3**
**Location**	49.26491, -0.87763	49.512660, 0.409829	48.851115,0.023732
**Previous crop**	*Beta vulgaris*	*Linum usitatissimum*	*Medicago sativa*
**Wheat cultivar**	Sacramento	Libravo	Chevignon
**Sowing date**	17-11-2018	16/10/2018	16-10-2018
**Organic matter input**	Yes	No	No
**Soil type**	Clay loams / Frank loams	Deep silt	Superficial clay limestone
**pH (water)**	6.2	7.3	8.4
**CEC (mol kg^{-1})**	9.5	8.5	10.8
**Organic matter (%)**	2.4	2.1	3.4
**C/N Ratio**	Not determined	8.1	7.6
**Total N (%)**	Not determined	0.15	0.26
**Organic C (%)**	1.42	1.22	1.98
**Available P (ppm)**	132	101	62
**Exchangeable K (ppm)**	168	238	254
**Exchangeable Mg (ppm)**	104	99	216
**CaO (‰)**	2.1	3.6	12.3
