# Supplementary material for: Biostimulant Effects of Glutacetine® and Its Derived Formulations Mixed With N Fertilizer on Post-heading N Uptake and Remobilization, Seed Yield, and Grain Quality in Winter Wheat
Source: Front Plant Sci. 2020 Nov 13;11:607615. doi: 10.3389/fpls.2020.607615 (PMC7691253; doi:10.3389/fpls.2020.607615)
Supplement: Supplementary file 2 [file Table_2.pdf]

Supplementary Table 2. Nitrogen management in the 3 field trials (Exp. 2)

	Site 1	Site 2	Site 3
**Humus effect**	65	50	30
**Previous crop effect**	20	0	40
**M.O. supply effect**	12	0	0
**N uptook**	10	29	26
**N residue (NO_3^-/NH_4^+)**	33 (25/8)	35 (33/2)	42 (34/8)
**Total N soil supply (kgN ha^{-1})**	140	114	138
**Crop needs**	272	304	270
**N remaining**	20	20	20
**Total needs**	292	324	290
**N fertilization**	152	210	152
**Supply 1**	61	100	62
**Supply 2**	31	50	30
**Supply 3**	60	60	60
