# Supplementary material for: Biostimulant Effects of Glutacetine® and Its Derived Formulations Mixed With N Fertilizer on Post-heading N Uptake and Remobilization, Seed Yield, and Grain Quality in Winter Wheat
Source: Front Plant Sci. 2020 Nov 13;11:607615. doi: 10.3389/fpls.2020.607615 (PMC7691253; doi:10.3389/fpls.2020.607615)
Supplement: Supplementary file 3 [file Table_3.pdf]

**Supplementary Table 3.** Analysis of variance of greenhouse experiment (Exp. 1)

| | | Treatment | Dose | Treatment*Dose |
|---|---|---|---|---|
| Figure 1 | Plant DW | | ** | |
| | Seed Yield | * | ** | |
| | Root DW | | | |
| | Straw DW | | ** | |
| | Grain number spike$^{-1}$ | | ** | |
| | Fruiting efficiency | *** | *** | * |
| Figure 2 | Total plant N | | | |
| | N Harvest Index | * | ** | |
| | Total grain N | | ** | |
| | %N in grain DW | | | |
| | %N in root DW | *** | *** | |
| | %N in straw DW | | * | |
| Figure 3 | Post-heading N uptake | *** | | |
| | Senescence speed | *** | *** | *** |
| | %N remobilized from roots | *** | *** | |
| | %N remobilized from straws | ** | *** | * |
| Figure 4 | Phytate content in grain | | | |
| | Phytate / Zn molar ratio | | | |
| | Phytate / Fe molar ratio | | | |
| Table 2 | Harvest index | * | | |
| | Spike number / plant | | | |
| | Grain number / plant | | * | |
| | 1000 seeds weight | * | | * |
| | Specific weight | | | |
| | Protein content | | | |
| | N Use Efficiency | | ** | |
| Table 3 | K content in grains | | | |
| | P content in grains | * | | |
| | S content in grains | | | |
| | Mg content in grains | | * | |
| | Ca content in grains | | | |
| | Na content in grains | | ** | |
| | Fe content in grains | | | |

| | | | | |
|---|---|---|---|---|
| | **Zn content in grains** | | | |
| | **Mn content in grains** | | | |
| | **Cu content in grains** | | * | * |
| | **Mo content in grains** | ** | | |
| | **B content in grains** | | | |
| | **Cd content in grains** | *** | | * |
| | **Se content in grains** | ** | | |
| | **Total N at heading** | *** | ** | |
| **Figure S2** | **N amount grain$^{-1}$** | | | |
| | **Grain N-to-S ratio** | | | |

*** : P < 0.001, ** : P < 0.01 and * : P < 0.05.
