# Supplementary material for: Biostimulant Effects of Glutacetine® and Its Derived Formulations Mixed With N Fertilizer on Post-heading N Uptake and Remobilization, Seed Yield, and Grain Quality in Winter Wheat
Source: Front Plant Sci. 2020 Nov 13;11:607615. doi: 10.3389/fpls.2020.607615 (PMC7691253; doi:10.3389/fpls.2020.607615)
Supplement: Supplementary file 4 [file Table_4.pdf]

Supplementary Table 4. Proteomics data

Accession	Description	Average Mass	Significance	Coverage (%)	#Peptides	#Unique	PTM	Control Intensity (top-3 peptides)	Glutacetine® Intensity (top-3 peptides)	Group Profile (Ratio)
GLT1_WHEAT	Glutenin high molecular weight subunit PC256 (Fragment)	10896	100.48	29	10	3	Oxidation (M), Acetylation (K), Carbamidomethylation, Deamidation (NQ), Propionamide	9.47E+03	3.85E+03	2.46:1.00
A0A0K2QJY6_WHEAT	Alpha/beta-gliadin	35863	75.17	32	6	2	Carbamidomethylation, Deamidation (NQ), Propionamide	4.78E+03	2.22E+03	2.16:1.00
A0A3B5XV32_WHEAT	12S seed storage globulin 1	63867	45.57	51	33	5	Acetylation (K), Carbamidomethylation, Deamidation (NQ), Propionamide	8.89E+03	4.97E+03	1.79:1.00
A0A3B6T7X8_WHEAT	Avenin-like b6	31911	36.11	62	20	9	Oxidation (M), Carbamidomethylation, Deamidation (NQ), Propionamide	1.85E+04	1.19E+04	1.56:1.00
A0A3B6ML15_WHEAT	Methylcrotonoyl-CoA carboxylase	81186	34.11	9	4	4	Acetylation (K), Deamidation (NQ)	2.43E+04	1.48E+04	1.64:1.00
A0A3B6LKD8_WHEAT	Clathrin heavy chain	193406	30.24	9	11	5	Carbamidomethylation, Deamidation (NQ)	6.16E+03	3.87E+03	1.59:1.00
A0A3B6TZD9_WHEAT	Basic endochitinase	28401	26.89	82	29	5	Oxidation (M), Acetylation (K), Carbamidomethylation, Deamidation (NQ), Propionamide	4.60E+03	3.00E+03	1.53:1.00
RBL_WHEAT	Ribule bisphphate carboxylase large chain	52851	25.23	11	4	2	Carbamidomethylation, Deamidation (NQ), Propionamide	1.09E+04	7.24E+03	1.51:1.00
AVLB1_WHEAT	Avenin-like b1	32727	21.2	45	7	3	Oxidation (M), Carbamidomethylation, Deamidation (NQ), Propionamide	1.20E+04	6.43E+03	1.87:1.00
GLT0_WHEAT	Glutenin high molecular weight subunit DY10	69629	20.71	42	21	6	Oxidation (M), Carbamidomethylation, Deamidation (NQ), Propionamide	4.73E+03	2.37E+03	2.00:1.00
A0A0K2QJX7_WHEAT	Alpha/beta-gliadin	35525	20.38	28	7	2	Carbamidomethylation, Deamidation (NQ), Propionamide	1.47E+04	7.20E+03	2.05:1.00
