# Supplementary material for: Biostimulant Effects of Glutacetine® and Its Derived Formulations Mixed With N Fertilizer on Post-heading N Uptake and Remobilization, Seed Yield, and Grain Quality in Winter Wheat
Source: Front Plant Sci. 2020 Nov 13;11:607615. doi: 10.3389/fpls.2020.607615 (PMC7691253; doi:10.3389/fpls.2020.607615)
Supplement: Supplementary file 5 [file Image_1.pdf]

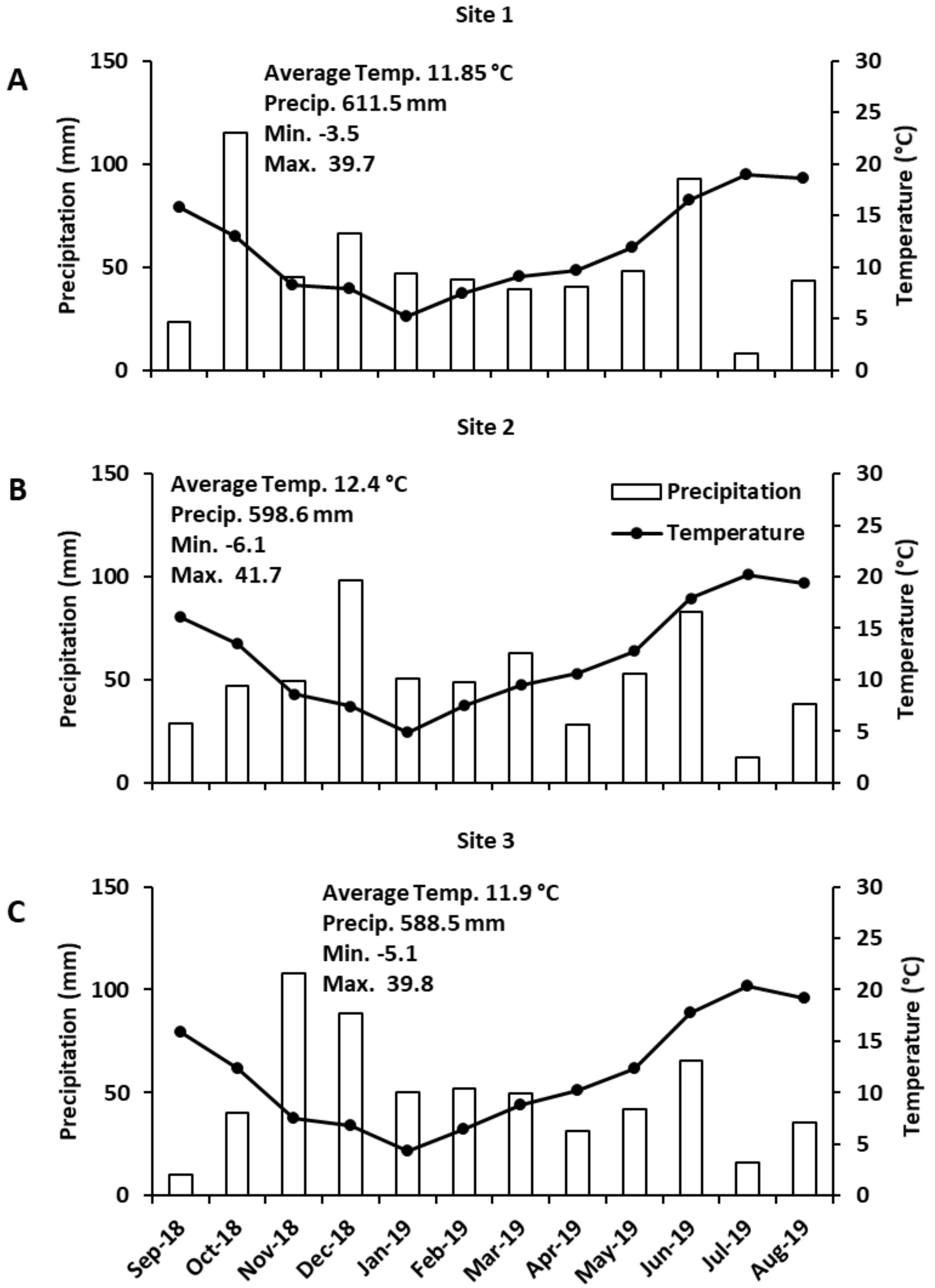

Supplementary Figure 1. Weather conditions between September 2019 and August 2020 in the 3 experimental sites. (A) Site 1, (B) Site 2 and (C) Site 3.
