# Supplementary material for: Biostimulant Effects of Glutacetine® and Its Derived Formulations Mixed With N Fertilizer on Post-heading N Uptake and Remobilization, Seed Yield, and Grain Quality in Winter Wheat
Source: Front Plant Sci. 2020 Nov 13;11:607615. doi: 10.3389/fpls.2020.607615 (PMC7691253; doi:10.3389/fpls.2020.607615)
Supplement: Supplementary file 7 [file Image_3.pdf]

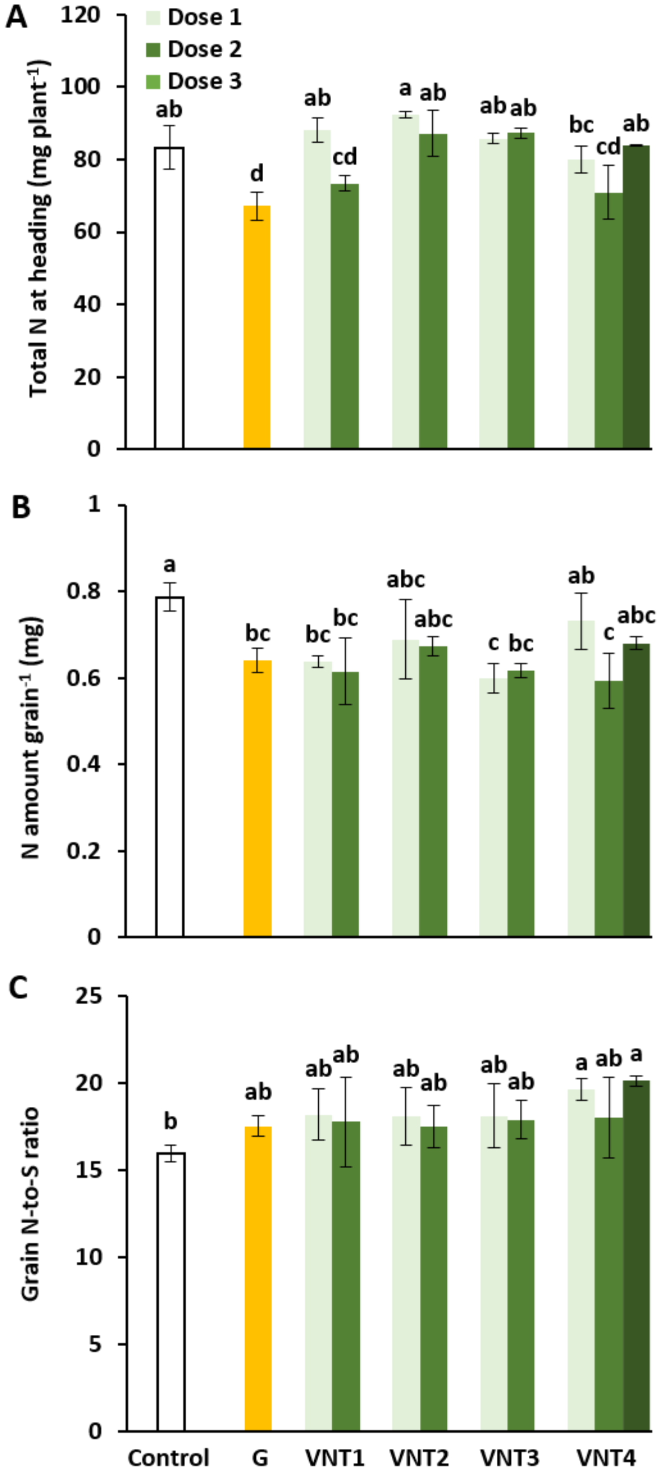
A
B
C
Dose 1
Dose 2
Dose 3
Total N at heading (mg plant-1)
N amount grain-1 (mg)
Grain N-to-S ratio
120
100
80
60
40
20
0
1
0.8
0.6
0.4
0.2
0
25
20
15
10
5
0
ab
d
ab
cd
a
ab
ab
ab
bc
cd
ab
a
bc
bc
bc
abc
abc
c
bc
ab
c
abc
b
ab
ab
ab
ab
ab
ab
ab
a
ab
a
Control
G
VNT1
VNT2
VNT3
VNT4

Supplementary Figure 3. Effect of biostimulant formulations mixed with N fertilizer on total N at heading, the N amount per grain and the grain N-to-S ratio in wheat (*Triticum aestivum* L.). (A) Total N at heading, (B) the N amount per grain, and (C) the Grain N-to-S ratio. Plant culture was carried out under semi-hydroponic conditions on a sand/perlite (1/1) substrate (see Materials and methods for details). N was provided at tillering (eq. 50 kgN ha^{-1}), the 2.5 cm head stage (eq. 80 kgN ha^{-1}) and heading (eq. 20 kgN ha^{-1}). Glutacetine® (G) was mixed with N fertilizer at the heading stage at a dose of 5 L ha^{-1} and applied as a foliar treatment. Four other formulations (VNT1, VNT2, VNT3 and VNT4, see Table 1 for composition of each) were mixed with N fertilizer at different doses: dose 1 was 83 mL kgN^{-1}, dose 2 was 166 mL kgN^{-1} and dose 3 was the equivalent of 3 x 5 L ha^{-1}. Dose 3 was tested only with VNT4. Plants were harvested at heading stage (GS59) and maturity (GS89). Bars indicate means ± SE. Different letters denote significant differences according to Fisher's test ($p < 0.05$; $n = 3$).
