# Supplementary material for: Biostimulant Effects of Glutacetine® and Its Derived Formulations Mixed With N Fertilizer on Post-heading N Uptake and Remobilization, Seed Yield, and Grain Quality in Winter Wheat
Source: Front Plant Sci. 2020 Nov 13;11:607615. doi: 10.3389/fpls.2020.607615 (PMC7691253; doi:10.3389/fpls.2020.607615)
Supplement: Supplementary file 8 [file Image_4.pdf]

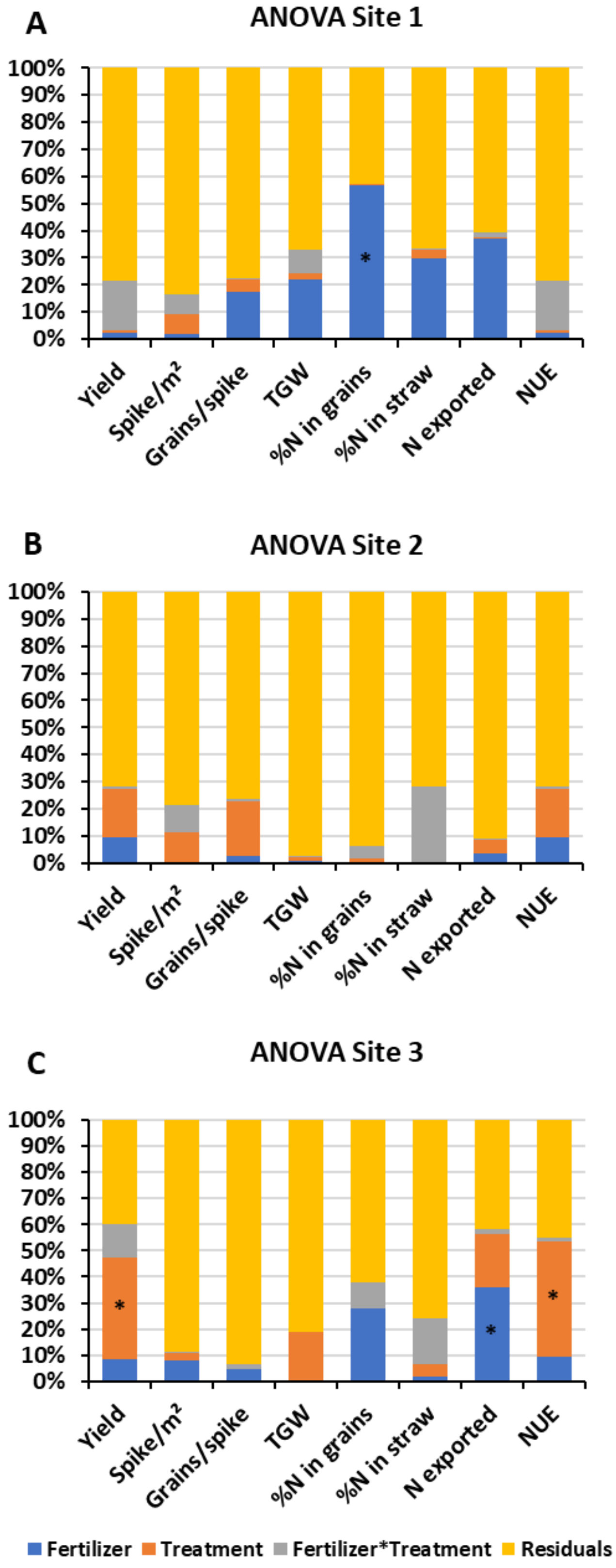
A
ANOVA Site 1
B
ANOVA Site 2
C
ANOVA Site 3
100%
90%
80%
70%
60%
50%
40%
30%
20%
10%
0%
*
Yield
Spike/m²
Grains/spike
TGW
%N in grains
%N in straw
N exported
NUE
Fertilizer
Treatment
Fertilizer*Treatment
Residuals

Supplementary Figure 4. Schematic representation of ANOVA of Yield (t ha^{-1}), Spike number per m^{-2}, Grain number per spike, thousand grain weight (TGW), N content in grains (%), N content in straw (%), N exported by grains (kg ha^{-1}) and NUE (kg kg^{-1}). (A) ANOVA Site 1 using data with the 2 fertilizers, (B) ANOVA Site 2 using data with the 2 fertilizers and (C) ANOVA Site 3 using with the 2 fertilizers. *** : P < 0.001, ** P < 0.01 and * : P < 0.05.
