# Supplementary material for: Biostimulant Effects of Glutacetine® and Its Derived Formulations Mixed With N Fertilizer on Post-heading N Uptake and Remobilization, Seed Yield, and Grain Quality in Winter Wheat
Source: Front Plant Sci. 2020 Nov 13;11:607615. doi: 10.3389/fpls.2020.607615 (PMC7691253; doi:10.3389/fpls.2020.607615)
Supplement: Supplementary file 9 [file Image_5.pdf]

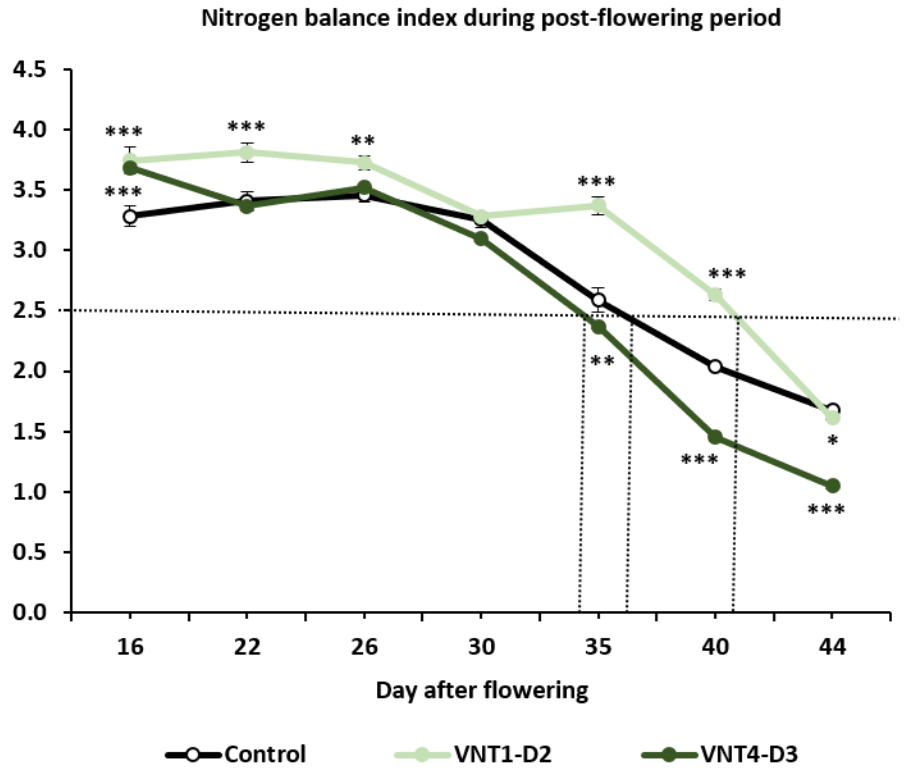

Supplementary Figure 5. Effect of biostimulant formulations mixed with N fertilizer on N balance index during post-flowering period in wheat (*Triticum aestivum* L.). N balance index of vegetative parts were measured during post-flowering period with an optical sensor system (fluorimeter Multiplex®, Force A, Orsay, France). A value below 2.5 indicates that the plant is in senescence (general yellowing of leaves). Plant culture was carried out under semi-hydroponic conditions on a sand/perlite (1/1) substrate (see Materials and methods for details). N was provided at tillering (eq. 50 kgN ha^{-1}), the 2.5 cm head stage (eq. 80 kgN ha^{-1}) and heading (eq. 20 kgN ha^{-1}). VNT1 and VNT4 (see Table 1 for composition of each formulation) were mixed with N fertilizer at different doses: dose 2 was 166 mL kg N^{-1} and dose 3 was the equivalent of 3 x 5 L ha^{-1}. Dose 3 was tested only with VNT4. Bars indicate means ± SE. Different letters denote significant differences according to Student's test ($p < 0.05$*, 0.01**, 0.001***; n = 4).
